# Preclinical efficacy of multi-targeting mRNA-based CAR T cell therapy in resection models of glioblastoma

**DOI:** 10.1016/j.omtn.2025.102676

**Published:** 2025-08-11

**Authors:** Oula K. Dagher, Martin Pedard, Darel Martinez Bedoya, Shawna K. Brookens, Denis Migliorini, Avery D. Posey

**Affiliations:** 1 Department of Systems Pharmacology and Translational Therapeutics, University of Pennsylvania Perelman School of Medicine, Philadelphia, PA 19104, USA; 2 Center for Cellular Immunotherapies, University of Pennsylvania Perelman School of Medicine, Philadelphia, PA 19104, USA; 3 Brain Tumor and Immune Cell Engineering Laboratory, AGORA Cancer Research Center, 1005 Lausanne, Switzerland; 4 Swiss Cancer Center Léman, 1005 Lausanne, Switzerland; 5 Center for Translational Research in Onco-Hematology, University of Geneva, 1211 Geneva, Switzerland; 6 Department of Oncology, University Hospitals of Geneva (HUG), 1205 Geneva, Switzerland; 7 Corporal Michael J. Crescenz VA Medical Center, Philadelphia, PA 19104, USA

**Keywords:** MT: Delivery Strategies, chimeric antigen receptor T cells, messenger RNA, glioblastoma, immunotherapy, multiple targeting, tumor-associated antigens, surgical resection, lipid nanoparticles, electroporation

## Abstract

Traditional viral-based chimeric antigen receptor (CAR) T cell therapies have vanquished multiple blood malignancies with decade-long remissions yet struggle against solid tumors. Nonviral engineering of CAR T cells via electroporation or lipid nanoparticle (LNP) delivery of CAR-encoding mRNA results in highly efficient yet transient CAR expression, challenging the adequacy of available preclinical models for mRNA-based CAR T cell evaluation. This study presents a unique three-pronged approach that combines mRNA-based CAR T cells, multi-targeting of glioblastoma (GBM)-associated receptors, and maximal surgical resection as a novel and readily translatable platform for preclinical evaluation of mRNA-based CAR T cells against solid tumors. We performed head-to-head *in vitro* and *in vivo* analyses of mRNA-based CAR T cells generated using different expansion conditions, mRNA delivery methods, or combination approaches. Besides potent *in vitro* cytotoxicity, our findings unveil a therapeutic window of anti-tumor efficacy, as well as robust and durable complete remissions in xenograft mouse models of GBM receiving maximal surgical resection and locoregional injections of multivalent CAR T cells (MVCAR). Such efficacies were significantly better in 5-day expanded versus quiescent T cells. Interestingly, MVCAR T cells were superior to pooled CAR T cells (CARPool) expressing the same CAR scFv combinations in an orthotopic resection model of GBM.

## Graphical abstract

**Figure:**
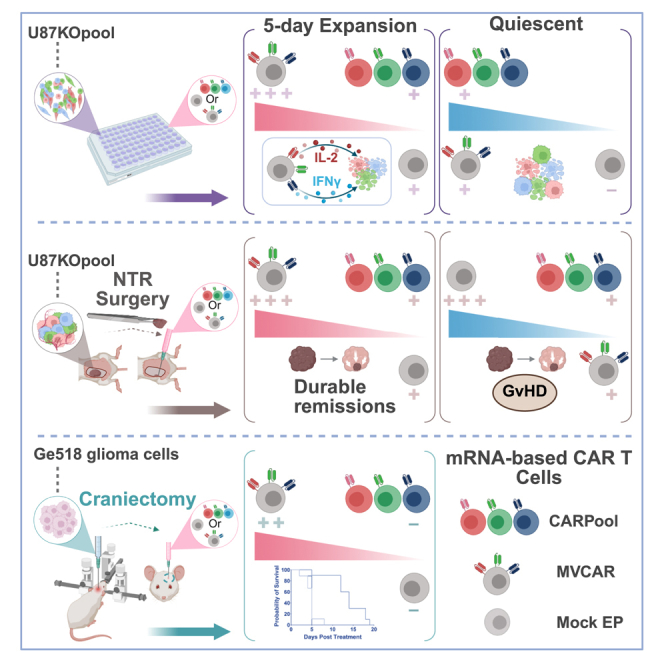

## Teaser

Dagher and –colleagues demonstrate that transient, multi-targeting mRNA-engineered CAR T cells, delivered after maximal glioblastoma resection, eradicate tumors in subcutaneous and orthotopic patient-derived xenograft models. This readily manufactured approach counteracts antigen heterogeneity and advances a safe, adaptable immunotherapy platform for otherwise incurable solid tumors.

## Introduction

Standard therapy for glioblastoma (GBM), the deadliest form of primary brain tumors, includes maximal surgical resection combined with adjuvant radio/chemotherapy.^1^ With the advent of chimeric antigen receptor (CAR) T cell therapy, mono- and multi-targeting CAR T cell therapies directed against GBM-associated antigens have been developed. IL13Rα2,^2^^,^^3^^,^^4^ EGFR and EGFR variant III (EGFRvIII),^5^^,^^6^^,^^7^^,^^8^ HER2,^9^^,^^10^ EphA2,^11^ CSPG4,^12^ B7-H3,^13^ and PTPRZ1^14^ are prominent GBM-associated membrane antigens (non-exhaustive list) that have been evaluated preclinically and/or clinically as CAR T cell targets in GBM. However, GBM-associated antigens are limited, and their heterogeneous expression—both within tumors and among patients—poses a major challenge to CAR T cell design and efficacy.^15^ Tumor antigen loss, immunosuppressive cues within the tumor microenvironment (TME) and stroma, hypoxic and nutrient depleting conditions, cancer stem cell renewal capacity that contributes to tumor heterogeneity and resistance to therapy, and limited CAR T cell infiltration and persistence within the TME are additional factors that limit the efficacy of immunotherapies against solid tumors, including GBM.^16^^,^^17^ To circumvent such challenges, multi-targeting and combinational approaches have been implemented.^6^^,^^18^^,^^19^^,^^20^^,^^21^^,^^22^

Traditional CAR T cell therapies, developed using viral or transposon-based CAR integration strategies, encounter several drawbacks. Examples include safety concerns such as the risk (albeit extremely rare) of insertional mutagenesis, oncogenesis, and host immune system activation; variability in CAR integration efficiency; limited cargo capacity; complex and expensive manufacturing; and complicated regulatory requirements.^16^^,^^23^ Compared with traditionally manufactured CAR T cells, mRNA-based CAR T cells offer multiple advantageous features. These include a high safety profile due to transient CAR expression, high CAR expression efficiencies, cost-effective and fast manufacturing, flexibility for multiplexing, and absence of complicated regulatory restrictions.^24^ Yet, there is a scarcity of studies that leverage mRNA-based CAR T cells against solid tumors, especially GBM,^14^^,^^25^^,^^26^ possibly due to hindering factors such as transient CAR expression and diminishing anti-tumor lysis by engineered T cells in bulky tumor masses. While traditional CAR T cells can persist in the blood of patients with hematologic malignancies, persistence is yet to be achieved in solid tumor settings. However, persistent CAR T cells tend to become exhausted and hypofunctional with time. Multi-dosing of transient CAR T cells, using RNA-based technologies, thus presents a viable option to circumvent dysfunctional CAR T cell conditions while maintaining anti-tumor efficacies within established therapeutic windows.^27^

Here, we propose a three-pronged platform for the preclinical assessment of locoregional administration of multi-targeting mRNA-based CAR T cells into maximally resected GBM xenografts or orthotopic tumors. Our findings demonstrate unprecedented distinguishable aspects and windows of efficacy of mRNA-based CAR T cells, generated using different combination approaches and expansion conditions.

## Results

### Screening for efficient mRNA-based CAR T cell combinations

**Figure 1:**
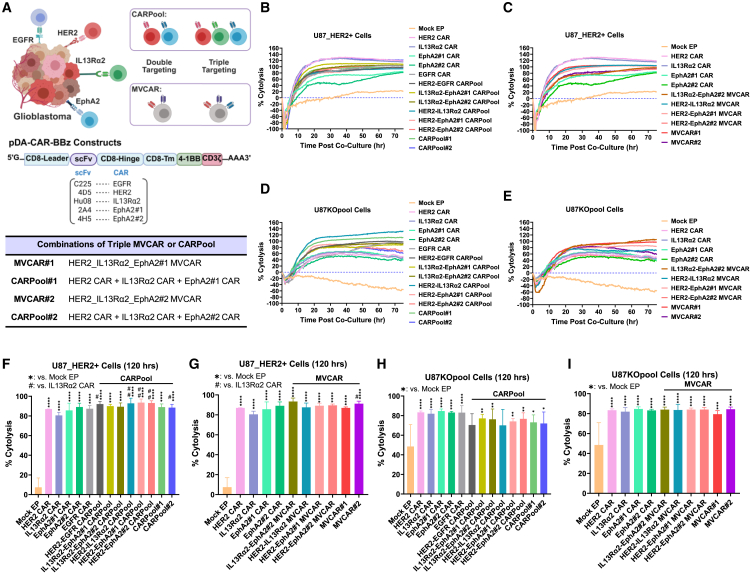
Efficient specific lysis of U87KOpool or U87_HER2+ cells with single, dual, and triple CARPool or MVCAR combinations of selected mRNA-based CAR T cells at 10:1 effector-to-target ratio (A) Study scope and CAR designs. Two distinct multi-targeting approaches were used: (1) pooled CAR T cells (CARPool), generated by electroporating each set of cells with one type of CAR-BBz-encoding mRNA, then pooling cells in equal ratios or (2) multivalent CAR T cells (MVCAR), generated by electroporating each set of cells with two or three CAR-BBz-encoding mRNAs to generate dual or triple MVCAR (nomenclature described in the table). (B–M) Quiescent T cells EP-ed with mRNA encoding for HER2 CAR, IL13Rα2 CAR, EphA2#1 CAR, EphA2#2 CAR, EGFR CAR, or combinations thereof were cocultured with U87_HER2+ or U87KOpool target cells at effector-to-target ratios of 10:1. (B–E) Normalized impedance-based cytolysis analysis of real-time data (mean % cytolysis calculated from *n* = 2 different experiments ran using T cells from two different healthy donors, with two biological replicates per sample). (F–I) Flow-based cytolysis analysis after 5 days of coculture (mean ± SD of % cytolysis calculated from *n* = 2 different experiments ran using T cells from two different healthy donors, with two biological replicates per sample). Different healthy donors were used to source CAR T cells for each cytotoxicity assay. (F–I) Ordinary one-way ANOVA, followed by Tukey multiple comparison analysis. ∗: vs. Mock EP; #: vs. IL13Rα2 CAR. ∗ or #: *p* < 0.05, ∗∗ or ##: *p* < 0.01, ∗∗∗ or ###: *p* < 0.001, ∗∗∗∗ or ####: *p* < 0.0001.

We prioritized the prominently studied receptors, HER2, IL13Rα2, EphA2, EGFR, and CSPG4 as CAR targets for our single-, dual-, and triple-targeting CAR T cell combinations, leveraging electroporation (EP)-mediated delivery of CAR-BBz-encoding mRNA (Figure 1A). We then prepared a set of U87-MG cell lines that lack HER2 (U87_HER2null) or express HER2 while lacking other antigens including EphA2 (U87_HER2+_EphA2KO), IL13Rα2 (U87_HER2+_IL13Rα2KO), EGFR (U87_HER2+_EGFRKO), or CSPG4 (U87_HER2+_CSPG4KO). While single-targeting CAR T cells demonstrated efficient antigen-dependent cytolysis, as well as human interferon gamma (IFN-γ) secretion in response to cognate antigen recognition on the surface of U87 cells, those CAR T cells failed to eradicate cognate antigen-depleted U87 cells (Figures S1A–S1D). Interestingly, however, U87_HER2null, U87_HER2+_EphA2KO, U87_HER2+_IL13Rα2KO, U87_HER2+_EGFRKO, and U87_HER2+_CSPG4KO cells pooled at equal ratios (U87KOpool) and were efficiently eradicated upon coculture with all tested single-targeting CAR T cells, comparable with specific lysis of U87-HER2+ cells that express all five targets (Figures S1A–S1C). These findings provide evidence of efficient target-mediated (CAR-dependent) and bystander killing, respectively, of pooled target-expressing and target-lacking U87 tumor cells (U87KOpool) by all single-targeting CAR T cells. Two distinct multi-targeting approaches were later used: pooled CAR T cells (CARPool) or multivalent CAR T cells (MVCAR), to generate dual or triple-targeting CAR T cell combinations (Figure 1A). A pilot screening of 28 different dual and triple CARPool or MVCAR combinations was performed (data not shown), and selected combinations with top-scoring cytotoxic potentials were further verified. Figure 1 shows cytolysis efficiencies as determined by impedance-based (Figures 1B–1E) and flow-based assays (Figures 1F–1I) following cocultures of multi-targeting CAR T cells with U87-HER2+ cells (Figures 1B, 1C, 1F, and 1G) or U87KOpool (Figures 1D, 1E, 1H, and 1I). To evaluate the type of resistant tumor cells surviving CAR T cell cytotoxicity, individual U87KO cell lines were stained with different combinations of CellTrace dyes prior to coculture with CAR T cells (Figures S2A–S2E). Given the considerable number of combination conditions evaluated, only a high effector-to-target (E:T) ratio of 10:1 was assessed here. Based on our findings (Figures S2A–S2E), EphA2KO, HER2null, and, to a lesser extent, IL13Rα2KO cells, were predominant within the treatment-resistant live cell populations, regardless of coculture combination settings and were thus chosen as targets for investigation in this study. To further investigate the differences in proliferation capacity between the chosen cell lines, we stained the cells with CFSE and monitored the changes in patterns of cell proliferation and population doublings in real time by eSight (Figure S2F), followed by counting the absolute live cells in each line at 119 h post-culture, by flow-based analysis (Figure S2G). We found that U87_HER2+_IL13RA2KO cells had the slowest growth patterns and highest doubling time of all tested groups. To limit the variability between our *in vitro* and *in vivo* assays, we used an equal ratio of each of U87_HER2null, U87_HER2+_EphA2KO, and U87_HER2+_IL13Rα2KO cell lines in all subsequent experiments in this study involving U87KOpool cells.

### Influence of expansion on mRNA-based CAR T cell cytotoxicity

**Figure 2:**
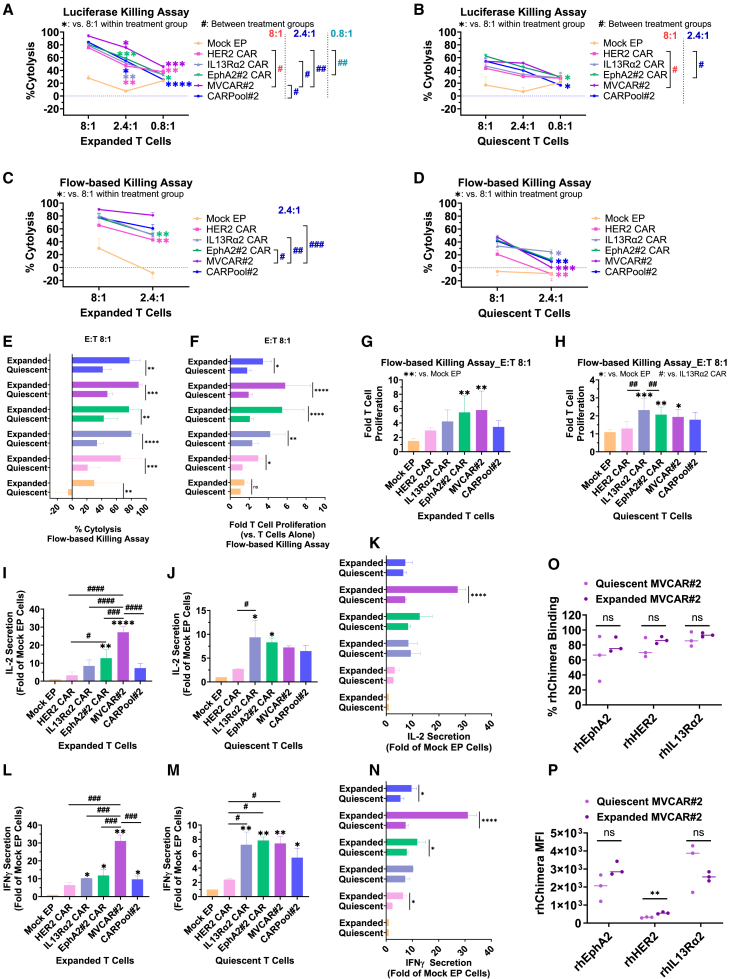
Influence of expansion on single versus multi-targeting mRNA-based CAR T cells T cells obtained from three different donors for each condition (expanded or quiescent) were Mock EP-ed, or EP-ed with mRNA encoding for HER2 CAR, IL13Rα2 CAR, EphA2#2 CAR, or combinations thereof (MVCAR#2). (IL-7/IL-15)-Conditioned medium was used in either condition of T cell expansion. In some instances, single-targeting CAR T cells were combined in equal ratios (CARPool#2). CAR T cells were then cocultured with U87KOpool-CBG-GFP/NLS cells at 8, 2.4, or 0.8 CAR+ cells:target cell ratios, respectively. A set of functional assays were performed: (A and B) mean ± SD luciferase-based cytolysis at 48 h post-coculture (three replicates per donor, three donors), (C–E) mean ± SD flow-based cytolysis, and (F–H) mean ± SD fold T cell proliferation measurements at day 5 post-coculture (three replicates per donor, two donors). (I–N) Mean ± SD fold of Mock EP T cell secretion of IL-2 and IFN-γ measured by ELISA 24 h post-coculture (*n* = 3 replicates per donor, at least two donors). (O and P) Flow cytometry staining with rhChimera compares staining efficiency and intensity between expansion groups (three different donors). (A–D, O, and P) Ordinary two-way ANOVA, post hoc Tukey analysis. (F, H–L, and N) Ordinary one-way ANOVA, post hoc Tukey. (M) Mixed-effect comparison analysis, post hoc Tukey. Symbols of significance are defined within graphs where necessary. ∗ or #: *p* < 0.05; ∗∗ or ##: *p* < 0.01; ∗∗∗ or ###: *p* < 0.001; ∗∗∗∗ or ####: *p* < 0.001.

Since prior T cell expansion is not a prerequisite for EP-mediated mRNA-based CAR T cell production, we performed head-to-head comparisons to investigate the *in vitro* cytotoxic efficacies of quiescent mRNA-based CAR T cells and their briefly 5-day expanded counterparts. To rule out which combination approach was favorable *in vitro*, we performed luciferase killing assays, flow-based killing assays, and enzyme-linked immunosorbent assays (ELISAs) on T cells from two to three different donors within each expansion group (Figures 2A–2N). Monovalent HER2 CAR, IL13Rα2 CAR, or EphA2#2 CAR T cells or triple combinations thereof (MVCAR#2 or CARPool#2) were prepared for each donor and cocultured with U87KOpool cells at three different E:T ratios of CAR+ T cells-to-target cells for 24 h (ELISA), 48 h (luciferase assay), or 120 h (flow-based assay) (Figures 2A–2N). Notably, there were no significant differences between quiescent and expanded MVCAR in terms of frequency or mean fluorescence intensity (MFI) of CAR expression, determined by individual recombinant human chimera (rhChimera) target antigen binding (rhEphA2, rhHER2, and rhIL13Rα2), although the conditions used for EP were different (Figures 2O and 2P). At high E:T ratios (8:1), expanded MVCAR had robust cytotoxicity (around 90% cytolysis efficiency), which was reproducible in both luciferase and flow-based killing assays (Figures 2A and 2C). Interestingly, at low E:T ratios (2.4:1), expanded MVCAR exhibited significant upregulation of human IL-2 and human IFN-γ cytokine secretions (Figures 2I and 2L) accompanied by significantly enhanced anti-tumor cytotoxicity as compared with monovalent CAR T cells (Figures 2A and 2C). While MVCAR T cells had significantly increased fold proliferation capacity as compared with Mock EP cells, upon coculture with U87KOpool cells, no significant difference in fold T cell proliferation was identified between MVCAR and monovalent CAR T cells (Figure 2G). Meanwhile, pooling CAR T cells in the expanded CARPool group did not result in any significant improvements in cytotoxicity, fold proliferation, or cytokine secretion when compared with single-targeting CAR T cells (Figures 2A, 2C, 2G, 2I, and 2L). As for quiescent MVCAR cells, an average of 50% cytolysis efficiency was obtained at high E:T ratios (8:1) and was consistent between the luciferase and flow-based killing assays (Figures 2B and 2D). Intriguingly, within the quiescent groups, CAR T cell combination approaches encompassing MVCAR or CARPool had no significant advantage over monovalent CAR T cells (Figures 2B, 2D, 2H, 2J, and 2M). When running head-to-head analysis between quiescent versus expanded CAR T cell groups, expanded cells outperform their quiescent counterparts in terms of cytotoxicity, fold proliferation, or cytokine secretion, as vividly seen within the MVCAR#2 groups (purple bars) (Figures 2E, 2F, 2K, and 2N).

### Effect of the extent of resection on CARPool and MVCAR cytotoxicity *in vivo*

**Figure 3:**
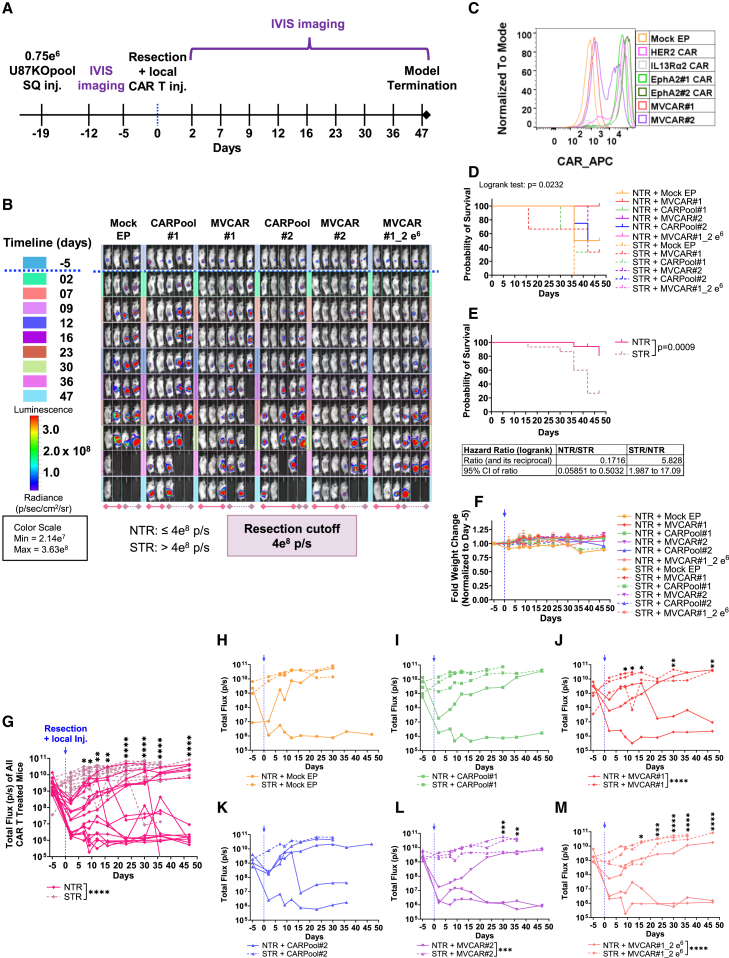
Influence of extent of resection on mRNA-based CAR T cell anti-tumor efficacy in GBM s.c. xenograft mice (A–C) U87KOpool-CBG+ cells were s.c. inoculated into the right flank of NSG mice and randomized into 6 groups (4–6 mice per group) based on BLI measurements before treatment. On day 19, mice received either STR (75% tumor resection) or NTR (90% tumor resection) surgeries and subsequent locoregional injection of Mock EP or mRNA-based multi-targeting CAR T cells, sourced from the same healthy donor (#TMP503). A resection cutoff that maintains minimal residual disease was determined as 4e8 p/s. (C) STR-operated mice had BLI > 4e8 p/s, while NTR-operated ones had BLI ≤ 4e8. (D) Mean ± SD fold change in body weight (two-way ANOVA, not significant) comparing STR- and NTR-operated mice in each group. (E) Kaplan-Meier survival analysis between groups. Log rank test for trend was significant (∗*p* = 0.0232) between all groups, but no significance was detected in pairwise comparison analyses by Holm-Sidak. (F) Kaplan-Meier survival analysis of pooled STR and NTR mice receiving CAR T cell treatment (log rank [Mantel-Cox] test, *p* = 0.009, as well as hazard ratios were calculated). (G–M) Longitudinal BLI measurements comparing pooled STR and NTR mice for all CAR T cell-treated mice (G) or STR and NTR mice per group (H–M), mixed effects multiple comparison analysis, post hoc Tukey. ∗: *p* < 0.05; ∗∗: *p* < 0.01; ∗∗∗: *p* < 0.001; ∗∗∗∗: *p* < 0.0001.

Given that temporary CAR expression in mRNA-based CAR T cells could limit cytotoxic intensity and durability against a bulky tumor mass, we hypothesized that leveraging maximal surgical resection and multiple-antigen targeting via locoregional administration of CARPool or MVCAR post-surgical resection would enable efficient and durable preclinical remissions. Clinically, the extent of resection (EOR), defined as the percentage of residual disease post-surgical operation, has been classified into subtotal reaction (STR), near-total reaction (NTR), or gross-total resection.^1^ We first aimed to study the influence of EOR on the anti-tumor efficacy of MVCAR or CARPool. Since operating surgical resections in the flank would enable easier and better control of EOR than in orthotopic resection models of GBM, we utilized a GBM xenograft mouse model and created a cutoff that maintains minimal residual disease to differentiate between STR- and NTR-operated mice (Figures 3A and 3B). Mice were subcutaneously (s.c.) inoculated with 0.75e6 U87KOpool-CBG+ cells, randomized into six groups based on the following treatments: Mock EP T cells, CARPool#1 (1e6 cells/mouse), CARPool#2 (1e6 cells/mouse), MVCAR#1 (1e6 cells/mouse), MVCAR#2 (1e6 cells/mouse), or MVCAR#1_2e6 (2e6 cells/mouse) (Figures 3B and 3C) and monitored for tumor growth. Regardless of the treatment provided, tumors in all STR-operated mice continued to grow exponentially, monitored until day 47 post-resection/injections (Figures 3A and 3B). However, NTR-operated mice had partial remission (PR) or complete remission (CR) in each of the MVCAR and CARPool treatment groups. This resulted in better survival rates and improved hazard ratios in NTR-operated mice receiving either CARPool or MVCAR as compared with STR-operated counterparts (Figures 3D and 3E). While a trend in weight changes could be seen starting at day 30 post-treatment, the low number of mice per group surviving at that time limited our statistical analysis and inference (Figure 3F). Remissions post-treatment were manifested by significantly lower total flux in NTR subgroups versus STR counterparts (Figures 3G–3M). For all animal models, CR was determined by total lack of luminescence upon IVIS imaging, that persisted longitudinally, and lack of bulk tumor mass by caliper measurements (not shown) or upon sacrifice.

### Functional assessment of quiescent CARPool and MVCAR in NTR-operated xenograft GBM-bearing mice

Given that minimally *ex-vivo*-expanded T cells are shown to have better persistence and central memory-like phenotype than those undergoing prolonged expansion,^28^ we repeated the previous model by locoregional injection of quiescent Mock EP, CARPool, or MVCAR T cells, sourced from healthy donor #ND637 (6 mice per group, Figure S3A) and incubated overnight in (IL-7/IL-15)-conditioned medium, to s.c. U87KOpool-bearing and NTR-operated mice. Among CAR T cell-treated groups, mice receiving MVCAR#2 had the best outcome with a 66.67% CR rate (4/6 mice) plus 13.33% PR. Surprisingly, we found that Mock EP-treated mice had 83.33% CR (Figure S3B). Overall, we realized that mice undergoing remission suffered from early signs of graft-versus-host disease (GvHD) that was occasionally lethal. No significant differences in the longitudinal weight changes or probability of survival were seen between treatment groups (Figures S3C and S3D). We then investigated whether locoregional injections of T cells would enable the systemic circulation required for durable immune responses. Flow cytometry staining of cells collected from peripheral blood and spleen detected more hCD4+ than hCD8+ cells in all specimens, regardless of treatment group (data not shown). However, the absolute count of human T cells in either blood or spleen obtained from CAR T cell-treated mice, was higher in non-responders versus responders (Figures S3E and S3F). Besides, more than 95% of hCD8+ in blood were double-negative for PD-1 and Tim-3 (PD1^−^/Tim-3^−^) (Figure S3E). However, in splenic T cells, the percent of PD1^−^/Tim-3^−^ cells slightly dropped in non-responders in favor of PD1^−^/Tim-3^+^ cells (Figure S3F). Interestingly, both hCD4+ and hCD8+ T cells were isolated from the primary tumor sites, with CD4/CD8 average ratios of 0.9732 (responders) and 1.859 (non-responders) (Figure S3G). Notably, there were no differences in the flow staining of resistant tumor cells for EphA2, IL13Rα2, or HER2 between treatment groups (Figure S3H). Mice that had metastasis were not included in the responders versus non-responders’ classification.

### Comparison of quiescent versus expanded T cell cytotoxicity in NTR-operated xenograft GBM-bearing mice

**Figure 4:**
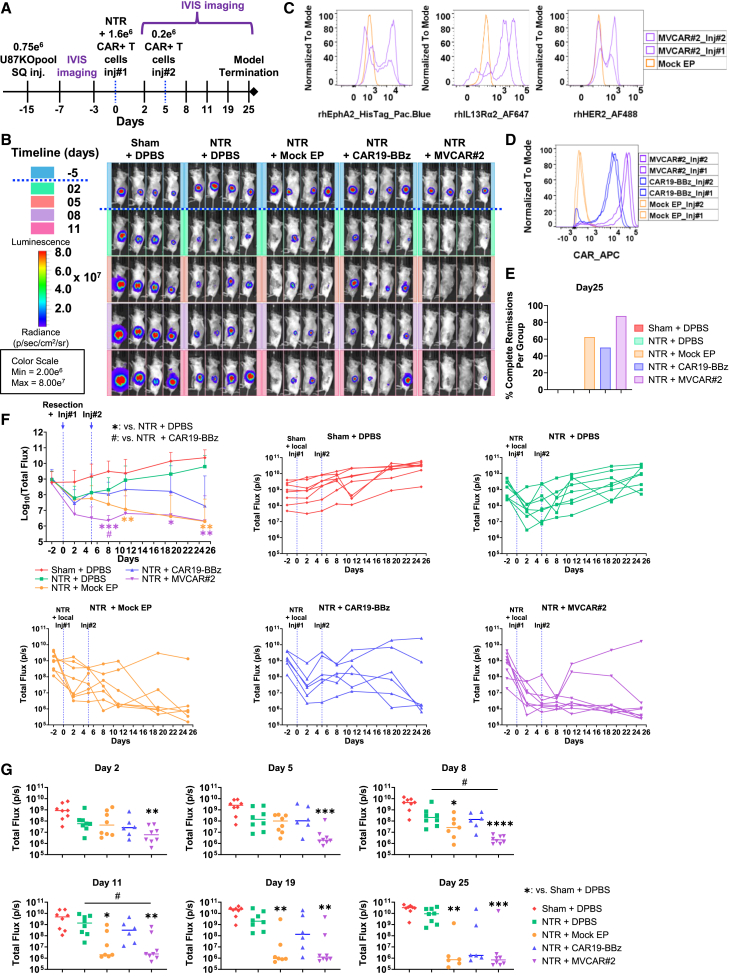
Investigating the efficacy of dual mRNA-based MVCAR T cell injections post-surgery in NSG mice bearing U87KOpool tumors (A–C) Mice bearing U87KOpool-CBG+ cells in the left s.c. flank were subjected, on day 0, to either sham operation or NTR operation and received locoregional DPBS, Mock EP, mRNA-based CAR19-BBz, or MVCAR#2 T cells, sourced from healthy donor #ND625. A second IVIS-assisted local injection was administered on day 5. All T cells were expanded in (IL-7/IL-15)-conditioned medium prior to injections. (A) Model timeline (eight mice per group except for CAR19-BBz, six mice). (B) Selected BLI images of four representative mice per group showing the CAR-dependent therapeutic window of activity of mRNA-based MVCAR T cells in comparison with other groups. (C and D) Flow histograms of CAR staining as well as staining with rhChimera for T cell groups used in both injections. (E) % CR per group on the day of model termination, day 25. Graphs in (F) show mean ± SD of log-transformed total flux data comparing all groups (mixed-effect comparison analysis, post hoc Tukey) as well as total flux of each mouse per group. (G) Side-by-side comparisons of changes in total flux between groups show CAR-dependent and CAR-independent windows of cytotoxicity at selected time points; Kruskal-Wallis ANOVA, post hoc Dunn’s multiple comparison analysis. ∗ or #: *p* < 0.05; ∗∗: *p* < 0.01; ∗∗∗: *p* < 0.001; ∗∗∗∗: *p* < 0.0001.

Having seen the high incidence of CR in mice treated with quiescent, (IL-7/IL-15)-conditioned Mock EP cells in the previous model, we next investigated the influence of expansion and/or IL-7/IL-15 preconditioning on the cytotoxicity of Mock or CAR-modified T cells, obtained from healthy donor #TMP518 in U87KOpool-s.c. inoculated and NTR-operated mice (5 mice per group) (Figure S4A). Consistent with data in Figure S3B, cytokine-conditioned quiescent Mock EP (Mock EP_Cyt_Quiescent) cleared the tumors in all mice within 23 days of treatment (100% CR) (Figures S4B and S3C; Figure 4D). NTD_quiescent T cells that were not conditioned in IL-7/IL-15 (Mock_NTD_Quiescent) showed same trend of CR, which indicates that IL-7/IL-15 pre-stimulation is not the main driver for such remissions. On the other hand, only (2/5) mice treated with expanded NTD cells (Mock_NTD_Expanded) showed signs of remission. Notably, mice treated with lentiviral (LV)-transduced EphA2#1 CAR T cells expanded in IL-7/IL-15-conditioned medium (EphA2#1 CAR_LV_Expanded T cells) had delayed onset of remissions (starting day 37) but reached 100% CR rate by the end of the model, day 56 (Figures S4B and S4C; Figure 4E). mRNA-based MVCAR#1-treated mice (MVCAR#1_EP_Cyt_Quiescent) had a similar incidence of CR (2/5 mice) to the one reported in the previous model (Figures S4B–S4E; Figure S3B). Notably, mice undergoing remissions within the quiescent groups had GvHD complications manifested in extreme weight loss (Figure S4F), hair loss, dehydration, lethargy, and death (Figure S4G). On the other hand, only one mouse receiving EphA2#1 CAR_LV_Expanded T cells had delayed onset of GvHD symptoms, and all the mice receiving EphA2#1 CAR_LV_Expanded T cells survived the treatment until the termination of the model. These data suggest that T cell-intrinsic CAR-independent killing mechanisms engage in the remissions seen in the NTD groups and might be partially related to the sheer number of locoregionally injected T cells as compared with remaining tumor cells post-NTR operation, reminiscent with what is seen with tumor-infiltrating lymphocytes (TILs).^29^ Such anti-tumor cytotoxicity was more potent in quiescent than *ex-vivo*-expanded T cells. These findings are consistent with a previous study that demonstrates superior anti-tumor efficacy in naive-derived adoptively transferred TCR-engineered T cells as compared with central memory-derived ones.^30^ However, additional studies are needed to decipher the exact mechanism of such potent cytotoxicity of quiescent T cells. As for LV-transduced CAR T cells, CAR-dependent cytotoxicity had a delayed onset but lasting efficacy with less pronounced off-tumor toxicities.

### Early therapeutic window of efficacy for MVCAR in NTR-operated xenograft GBM-bearing mice

Uncovering the therapeutic window of efficacy of MVCAR is critical for designing proper dosing timelines that would guarantee durable and robust remissions of mRNA-based CAR T cells. Here, we investigated the therapeutic window of efficacy in U87KOpool-s.c.-inoculated and NTR-operated mice receiving two locoregional doses, on day 0 and day 5, of expanded and (IL-7/IL-15)-preconditioned Mock EP, mRNA-based CAR19-BBz, or MVCAR#2. We also included two groups of either sham-operated or NTR-operated mice receiving saline (DPBS [Dulbecco’s phosphate-buffered saline]) (8 mice per group). Findings from these groups demonstrate that, while NTR operation slows tumor progression in mice, it is not sufficient to achieve CR alone, as opposed to groups receiving NTR+ CAR T cells (Figures 4A–4E). Notably, mice receiving MVCAR#2 had early onset of robust CR (87.5% rate) spanning a 7-day therapeutic window (between days 2 and 8) post-NTR that was durable until the termination of the model (day 25) (Figures 4B and 4E–4G). On the contrary, while 62.5% Mock EP-treated mice achieved CR on day 25, the onset of remission was delayed (started day 8), suggestive of distinct kinetics and mechanisms of action between CAR-dependent and CAR-independent T cell-intrinsic cytotoxicity. While mice treated with the irrelevant CAR19-BBz initially underwent additional tumor progression, a delayed onset of remissions, concomitant with the time of loss of CAR19 expression (around day 8) was realized, whereby 50% of mice had CR by day 25. These findings infer two subsequent mechanisms of killing in the mRNA-based CAR T cell-treated mice as seen in MVCAR#2: the first is CAR-dependent with early onset and robust killing efficiency, and only occurs when MVCAR#2 cells recognize the CAR targets on tumor cells, followed by CAR-independent T cell-intrinsic killing after day 8, once CAR expression is expected to be significantly diminished or lost (Figure S1E). Since CAR19-BBz CAR T cells do not specifically target U87 tumor cells, only the CAR-independent mechanisms were observed.

### LNP-based MVCAR production and *in vitro* verification

**Figure 5:**
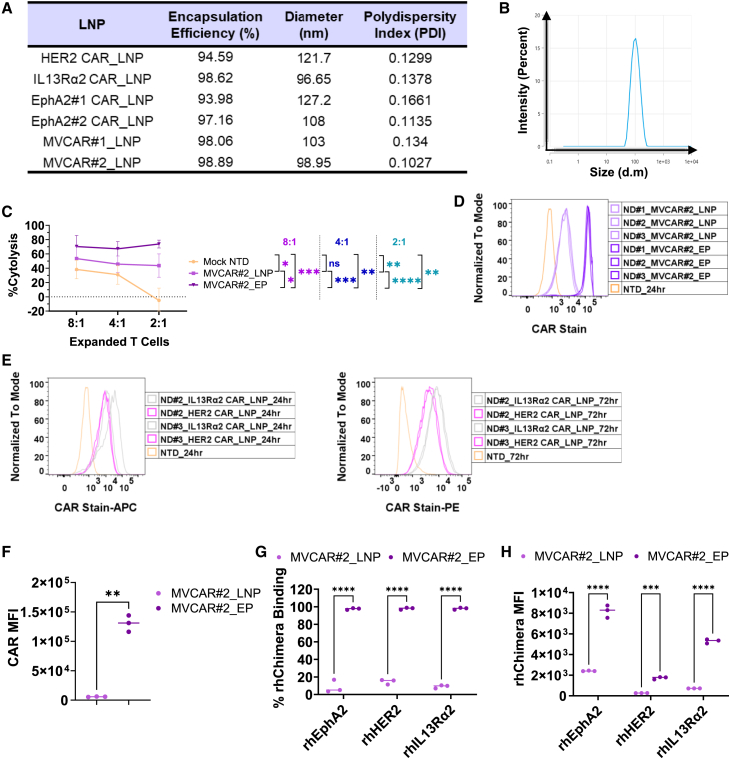
*In vitro* verification of LNP-based mRNA delivery on mRNA-based CAR T cell cytotoxicity The table in (A) provides physical characterization parameters regarding mRNA encapsulation efficiency, diameter, and PDI of single-CAR_LNP versus MVCAR_LNP batches. A representative size (z-average) distribution of MVLNPs is shown in (B), revealing a diameter of approximately 103 nm using dynamic light scattering. (C) T cells from three different donors (ND#1, #2, and #3) were used to investigate the cytotoxicity of LNP-delivered mRNA-based MVCAR#2 (MVCAR#2_LNP) compared with EP-ed MVCAR#2 (MVCAR#2_EP) at three different CAR+ cell-to-target cell ratios (E:T of 8:1, 4:1, and 2:1, respectively) using a flow-based killing assay, 5 days post-coculture with U87KOpool cells (mean ± SD of % cytolysis from *n* = 3 different donors with three biological replicates each, two-way ANOVA, post hoc Tukey). (D) Flow cytometry analysis of total CAR expression in EP- versus LNP-delivered MVCAR#2 from three different donors. Flow histograms in (E) show the stability of CAR expression in LNP-delivered mRNA-based HER2 CAR or IL13Rα2 CAR from two donors, up to 72 h post-LNP addition. Comparative analysis of rhChimera MFI (F, paired two-tail t test) and % binding (G and H, two-way ANOVA, post hoc Tukey) is provided for MVCAR#2_EP versus MVCAR#2_LNP. ∗: *p* < 0.05; ∗∗: *p* < 0.01; ∗∗∗: *p* < 0.001; ∗∗∗∗: *p* < 0.0001.

Lipid nanoparticle (LNP) formulation and delivery of mRNA is a rapidly advancing area within biotech and therapeutics industries. Here, we aimed to assess LNP-mediated delivery of mRNA-based CAR T cells *in vitro*. Single or triple CAR-encoding mRNA-loaded LNPs were formulated using the ionizable lipid mix (GenVoy-ILM Ignite) and the NanoAssemblr Ignite microfluidic device (refer to materials and methods for details on LNP formulation). Figures 5A and 5B lists the physical characteristics of mRNA encapsulation efficiency, size, and polydispersity index (PDI) of all formulated single CAR-BBz- or triple CAR-BBz (MVCAR)-loaded LNPs and shows the size (z-average) distribution of a representative sample of LNP-derived mRNA-based MVCAR (MVCAR#2_LNP). We next aimed to optimize the cytokine preconditioning and mRNA amounts for efficient LNP-based MVCAR. MVCAR#2_LNP expanded in serum-free ImmunoCult-XF medium supplemented with either 40 IU rhIL-2 or 5 ng/mL of each of human IL-7 and IL-15 were generated using either 1 or 3 μg of encapsulated mRNA per 1e6 T cells. Comparable specific lysis efficiency was achieved with MVCAR#2_LNP cells prepared by either mRNA-LNP doses, and regardless of cytokines used (Figures S5A–S5D).

To investigate the *in vitro* cytotoxic efficiency of MVCAR#2_LNP at multiple E:T ratios, we prepared MVCAR#2_LNP cells, using 1 μg encapsulated mRNA per 1e6 T cells, in ImmunoCult-XF medium supplemented with IL-7/IL-15 and compared with MVCAR#2_EP as a positive control. Our findings show that MVCAR#2_ LNPs had more than 50% cytolysis efficiency after 5 days of coculture with U87KOpool cells, which was maintained even at low (2.4:1) E:T ratios (Figure 5C). Furthermore, flow cytometry staining showed stable CAR expression that lasted for more than 72 h post-LNP addition (Figures 5D and 5E), which reflects the durability of mRNA-based CAR expression using this LNP delivery system and is reproducible with data recently published in Kitte et al.^31^ It was difficult to determine if MVCAR#2_EP outperformed MVCAR#2_LNP in our settings, given the differences in the total amount of mRNA delivered per 1e6 T cells in each route (1 μg total of multiplexed mRNA encoding for HER2 CAR, IL13Rα2 CAR, and EphA2#2 CAR in MVCAR#2_LNP versus 3.1 μg total [1 μg HER2 CAR: 1 μg IL13Rα2 CAR: 1.1 μg EphA2#2 CAR]) of the same combination of mRNA for MVCAR#2_EP. As mentioned in the materials and methods, we had optimized the amount of mRNA used for MVCAR#2_EP and used this amount for all the experiments included in this study. However, significant differences in the CAR MFI as well as percent binding and MFI of rhIL13Rα2, rhHER2, and rhEphA2 chimera were observed between MVCAR#2_LNP and MVCAR#2_EP cells (Figures 5F–5H).

### Efficient LNP-delivered MVCAR cytotoxicity in NTR-operated xenograft GBM-bearing mice

**Figure 6:**
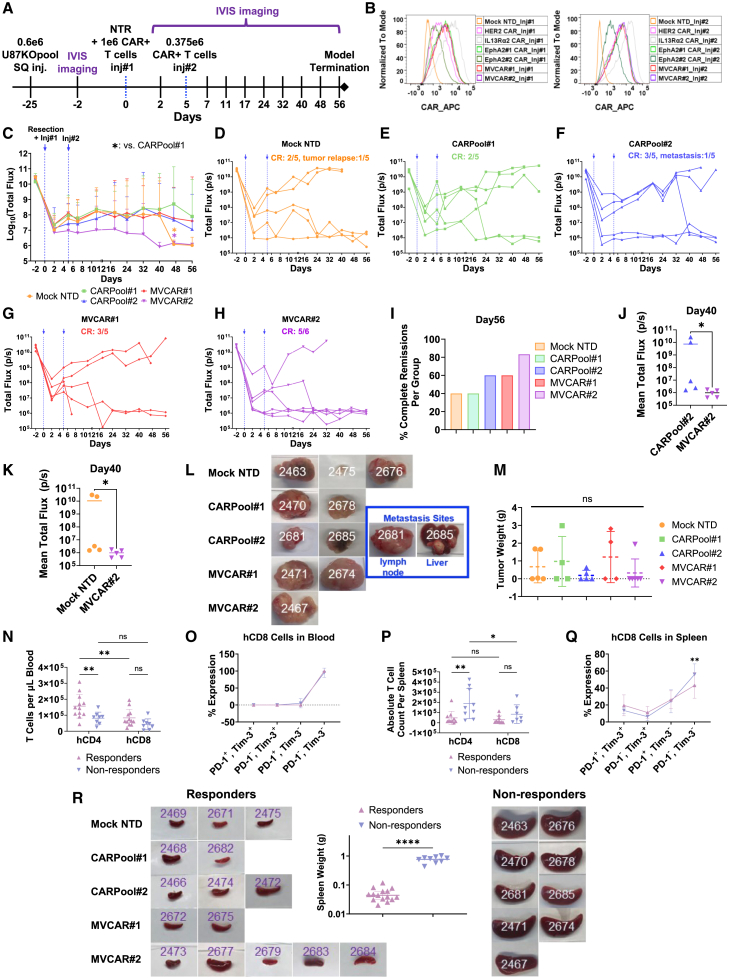
Preclinical evaluation of dual locoregional injections of Mock NTD or LNP-mediated mRNA-based MVCAR or CARPool T cells in NTR-operated mice bearing U87KOpool tumors Mice bearing U87KOpool-CBG+ cells in the left s.c. flank were subjected, on day 0, to NTR operation and received locoregional Mock NTD, CARPool, or MVCAR T cells, all sourced from healthy donor #ND410. A second IVIS-assisted local injection was administered on day 5. All T cells were expanded in IL-7/IL-15-conditioned medium and received Mock NTD or LNP-encapsulated mRNA encoding CAR-BBz. (A) Model timeline. (B) Flow histograms of CAR staining for T cells prepared by LNP delivery of mRNA and used in both injections. (C) Mean ± SD of log-transformed total flux (mixed-effect comparison analysis, post hoc Tukey). (D–H) Individual longitudinal total flux measurements. (I) % CR per group. (J and K) Selected dual comparisons of mean ± SD of BLI measurements on day 40 corresponding to MVCAR#2 versus CARPool#2 (J) or MVCAR#2 versus Mock NTD (K), Mann-Whitney U test. (L) Images of primary and metastatic tumors per mouse, collected during sacrifice. (M) No significant differences were found in weight of tumors collected from primary sites (Kruskal-Wallis ANOVA). (N–Q) Comparison analyses of mean ± SD of human CD4 and CD8 subset distribution and Tim-3 and/or PD-1 expression in T cells collected from the blood (N and O, collected on day 40) and spleen (P and Q, obtained on day of sacrifice) of responders versus non-responders within the pooled CAR T cells-treated mice. (N and P) Mixed-effect multiple comparison analysis, based on uncorrected Fisher’s LSD. (O and Q) Mixed-effect multiple comparison analysis, post hoc Tukey. (R) Images of spleens showing different spleen sizes in responders versus non-responders, as well as a plot of matching spleen weights (unpaired two-tail t test). ∗: *p* < 0.05; ∗∗: *p* < 0.01; ∗∗∗∗: *p* < 0.0001.

Given the durability of CAR-encoding mRNA expression seen with LNP delivery *in vitro* and documented elsewhere,^31^ we conducted side-by-side *in vivo* analysis of the cytotoxic potential of CARPool_LNP or MVCAR_LNP T cells, expanded in (IL-7/IL-15)-conditioned ImmunoCult-XF medium. NSG mice, bearing xenograft U87KOpool tumors, received NTR surgery and two locoregional injections of T cells, on days 0 and 5 post-NTR (5–6 mice per group) (Figures 6A–6H). For MVCAR#2-treated mice, we recapitulated the early onset 7 day therapeutic window of efficacy (at days 2–8 post-NTR) (Figures 6C and 6H), matching the efficacy of MVCAR#2_EP cells observed in Figure 4. The group receiving MVCAR#2 had the best outcome with an 83.33% CR rate, while CARPool#2 and MVCAR#1 demonstrated comparable outcomes at 60% CR (Figures 6C–6I). On day 40, MVCAR#2 anti-tumor efficacy outperformed CARPool#2 (Figure 6J) and Mock NTD (Figure 6K), reminiscent with *in vitro* observations. Although initially 3/5 mice receiving Mock NTD cells had tumor clearance, mouse no. 2475 started to undergo tumor relapse around day 40 (Figures 6D and 6L). Notably, mouse no. 2684 in the MVCAR#2 group had lymph node metastasis at early days post-NTR but was able to eventually sustain durable CR starting day 18 until the model termination (Figure 6H). However, both non-responder mice (nos. 2681 and 2685), who received CARPool#2 injections, had uncontrollable metastasis in the lymph node and liver, respectively (Figures 6F and 6L). Clinically, while the incidence of extracranial GBM metastasis is rare, it is more prominent following surgical procedures, and has been documented both in lymph nodes and liver, besides other sites.^32^^,^^33^^,^^34^ However, given that our model utilizes surgical incisions in a xenograft s.c.-based model, such incidence of metastasis is more likely to be higher. There was no significant difference between treatment groups in the mean weight of tumors collected at days of sacrifice from primary tumor sites (Figure 6M). For better analysis of the data, we pooled and classified all the specimens collected from mice receiving CAR combination therapy into responders or non-responders (Figures 6N–6R). Flow cytometry staining was performed on blood samples collected on day 40 and splenic T cells homogenized on day of sacrifice (Figures 6N–6Q). Human CD4 T cells were significantly higher in peripheral blood of responders than non-responders, and significantly higher than CD8, with peripheral blood CD4:CD8 ratio around 2:1 regardless of response group (Figure 6N). Most CD8 cells were PD-1^−^/Tim-3^−^ (Figure 6O), reminiscent with observations of quiescent CAR T cells (Figure S3E). On the other hand, all non-responders, regardless of treatment group, had splenomegaly (Figure 6R). Similar to the observations seen with the spleens of mice receiving quiescent T cells (Figure S3F), the splenic count of CD4 T cells was higher in non-responders, echoing the splenomegaly recorded in this group (Figures 6P and 6R). In splenic T cells, the percent of PD1^−^/Tim-3^−^ cells was slightly higher in non-responders (60%) than responders (40%), with no significant differences in PD1^+^, Tim-3^+^, or PD1^+^/Tim3^+^ cells (Figure 6Q). One mouse in each of the MVCAR#1 (no. 2675)- and MVCAR#2 (no. 2677)-treated groups had delayed onset of CR, accompanied by medium spleen size and elevated T cell count. Of all mice, only one mouse (no. 2677) within MVCAR#2 group had delayed onset of mild GvHD signs, mainly hair loss and dehydration, at day 56.

### Efficient cytotoxicity of expanded MVCAR#2 cells against patient-derived glioma stem cells *in vitro*

We next sought to investigate the cytotoxic potential of our mRNA-based single-targeting (HER2 CAR, IL13Rα2 CAR, or EphA2 CAR) and multi-targeting (CARPool#2 and MVCAR#2) CAR T cells against patient-derived glioma cells. Three patient-derived glioma stem cell (GSC) lines (nos. 5077, 8977, and 8979) were phenotypically evaluated for the expression of cell surface expression of HER2, IL13Rα2, and EphA2 against FMO controls (Figure S6A). All three GSC lines had subtle levels of HER2 expression, but considerable expression of IL13Rα2 and EPhA2 that varied between lines. Mock EP or CAR T cells were expanded for 5 days in (IL-7/IL-15)-conditioned medium prior to coculture with GSC lines at multiple low E:T ratios (1:1, 0.3:1, and 0.1:1) (Figure S6B). Our findings demonstrate cytotoxic efficacy of single-targeting mRNA-based CAR T cells reminiscent with the expression level of target antigens on the surface of GSC lines tested, with limited cytotoxic potential of HER2 CAR against all three GSC lines. Interestingly, however, multi-targeting of the same GSC lines with CARPool#2 or MVCAR#2 led to significantly increased cytotoxicity that was evident in two of the three GSC lines tested as compared with Mock EP cells or HER2 CAR T cells (GSC_5077 and GSC_8979).

### Expanded MVCAR#2 cells delay tumor growth in an intracranial orthotopic resection model of GBM

**Figure 7:**
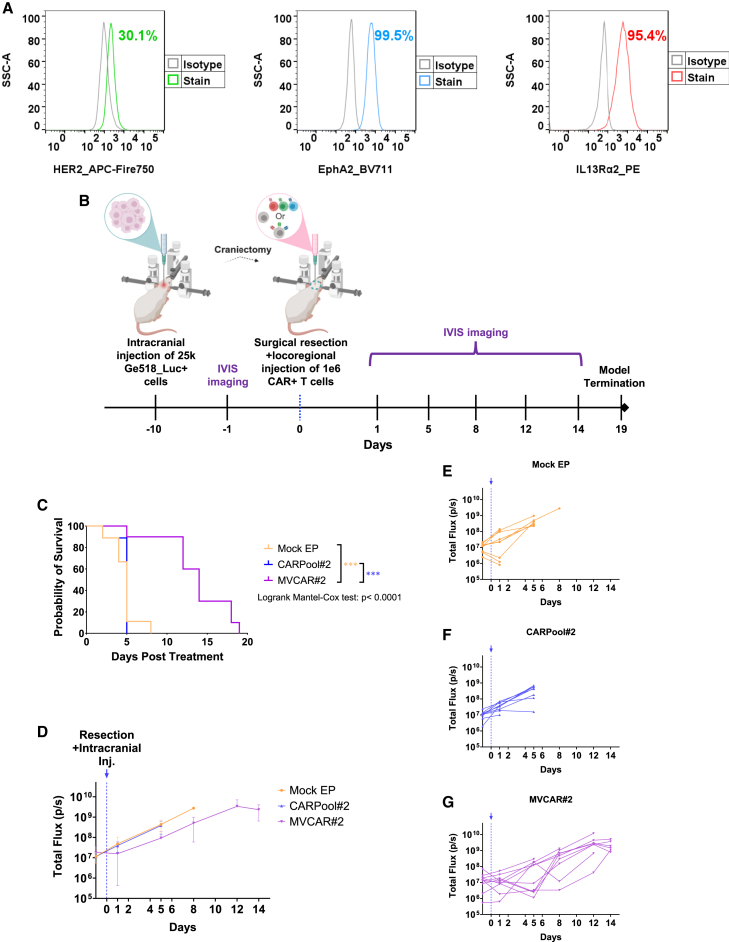
Preclinical evaluation of locoregional intracranial injections of Mock EP or EP-mediated mRNA-based MVCAR#2 or CARPool#2 T cells in an intracranial resection model of GBM-bearing patient-derived Ge518 tumors (A) Ge518 cells were phenotypically evaluated for cell surface target expression by staining with antibodies against HER2, IL13Rα2, and EphA2 prior to analysis by flow cytometry. (B–G) A total of 25,000 Ge518 cells was intracranially injected into NSG mice and monitored by IVIS imaging. On day 10, Ge518-bearing mice underwent craniectomy followed by locoregional injection of Mock EP, CARPool#2, or MVCAR#2 T cells that were expanded for 5 days in (IL-7/IL-15)-conditioned medium (9–10 mice per group). (B) Timeline of intracranial inoculation of Ge518 tumors as well as resection and locoregional injection of one dose of CAR T cells. (C) Kaplan-Meier survival analysis, log rank (Mantel-cox) test, followed by Holm-Sidak pairwise analysis. (D–G) BLI measurements plotted as mean ± SD total flux (D) or individually per mouse group (E–G). ∗∗∗: *p* < 0.001.

To evaluate the translational relevance of our platform, we validated the cell surface expression of HER2, IL13Rα2, and EphA2 in a patient-derived GBM cell line (Ge518) (Figure 7A), which we then employed in an orthotopic resection model of GBM (Figures 7B–7G). NSG mice were inoculated intracranially with 25,000 Ge518_Luc+ cells and monitored for 10 days (9–10 mice per group). On day 10, intracranial resection was performed followed by locoregional injection of one dose of 1e6 CAR+ cells of CARPool#2 or MVCAR#2, or its equivalence of Mock EP cells. Notably, 9/9 of CARPool#2-treated mice succumbed to death within 5 days of treatment, comparable with Mock EP-treated mice (8/9 mice died by day 5 and only 1 mouse survived to day 8 post-treatment) (Figures 7C–7F). Intriguingly, however, MVCAR#2 treatment delayed the tumor growth and significantly prolonged the overall survival of mice up to 19 days post-treatment, thus increasing the survival rate of mice in the Mock EP group by 57.89% and in the CARPool#2 group by 73.68% (Figures 7C, 7D, and 7G). Given the aggressive nature of the inoculated tumor and that only one dose of MVCAR#2 cells with transient CAR expression was administered intracranially, no CR was noticed. Although both CARPool#2 and MVCAR#2 T cells target the same triple antigens on tumor cells, our findings indicate that the multi-targeting approach for mRNA-based CAR T cells plays a critical role in the cytotoxic efficiency of such cells, and reproduces the superior efficacy seen with MVCAR#2 in the xenograft resection models discussed earlier.

## Discussion

This study presents a unique three-pronged approach that combines mRNA-based CAR T cells, multi-targeting of GBM-associated receptors, and NTR surgery as a new strategy to combat tumor heterogeneity and antigen loss, limit postoperative relapse, as well as achieve safe durable remissions. Our work further establishes a simple translational platform for preclinical evaluation of mRNA-based CAR T cells against resectable solid tumors. By allowing a heterogeneous population of tumor cells (U87KOpool) to engraft and grow to palpable sizes followed by NTR surgery that maintains minimal residual disease, we aimed to mimic the clinical scenario of tumor progression, heterogeneity, and clinical NTR operative procedures. The idea of combining surgical resection and locoregional injections of viral-transduced CAR T cells has been previously investigated for other solid tumors.^35^ However, the significance of our work is that it allowed the identification of a 7-day early onset therapeutic window of activity of mRNA-based MVCAR#2 that was only detectable in NTR operative settings, thus unveiling the potential of achieving durable CR using mRNA CAR T cells in xenograft models. Notably, in our orthotopic resection model, locoregional injection of only one dose of MVCAR#2 T cells directly post-resection significantly prolonged the survival of patient-derived tumor (Ge518)-bearing mice, suggestive of possible therapeutic translational advantage when using this platform with multiple locoregional MVCAR#2 injections. Additional translational significance of our work lies in the proposed concomitant locoregional injections of MVCAR directly post-NTR for complete eradication of any residual tumor cells post-surgical intervention, in newly diagnosed GBM patients. While this might be challenged by strict regulations mandated by the Food and Drug Administration, it is still feasible. In fact, in an ongoing phase 2b clinical trial (NCT04485949), newly diagnosed GBM patients receive biodiffusion chambers of IGV-001, an autologous cell immunotherapy with antisense oligonucleotide, 2 days post-surgical resection. Moreover, locoregional injection of multiple doses of multi-targeting and virally transduced CAR T cells has resulted in unprecedented radiographic regressions and transient remissions in recent clinical trials against GBM,^5^^,^^6^ which further indicates the translational feasibility of locoregional delivery of multiple infusions of CAR T cells.

Using our platform, it is evident that MVCAR outperforms CARPool both *in vitro* and *in vivo*. This is especially evident in the orthotopic resection model, where the overall survival rate was significantly improved in MVCAR#2-treated mice by 73.68% compared with CARPool#2. Moreover, our MVCAR T cells established efficient preclinical efficacy against CRISPR-edited heterogeneous pool of U87-MG cells (U87KOpool) as well as patient-derived GSC samples, both *in vitro* and *in vivo*. Various previous reports demonstrated the efficacy of tandem CAR T cells against GBM, including a trivalent tandem CAR that targets the same receptors assessed in this study, but with different CAR scFv combinations.^21^^,^^22^^,^^36^ Notably, our findings of the efficacy of triple-targeting MVCAR reproduces previously established efficacy against primary patient samples using a trivalent tandem CAR that targets the same receptors assessed in this study, but with different CAR scFv combinations.^36^ Using flow cytometry staining with rhChimera of target GBM receptors, we were able to identify population diversity within MVCAR (Figure S7). We believe that such population diversity brings in favorable outcomes in terms of MVCAR binding and cytotoxicity. Notably, bystander killing of target-lacking cells was documented with our monospecific CAR T cells when cocultured with U87KOpool cells. Diverse mechanisms of bystander killing have been reported in previous studies of CAR T cell therapies against GBM.^14^^,^^37^^,^^38^^,^^39^ While such mechanisms need further exploration for our CAR combinations, the advantage of bystander killing is more prominent in MVCAR, with the demonstrated population diversity, where both direct CAR-mediated as well as bystander killing capacity could be exerted by each subpopulation against tumor cells lacking one or more CAR targets. However, all our *in vivo* models were operated in NSG mice that lack a competent immune system, which limits our understanding of the possible interactions of MVCAR with other immune cell components including the immunosuppressive TME that is maintained in syngeneic mice. Future directions include conducting additional experiments that explore the safety of MVCAR in syngeneic immunocompetent mouse models of GBM.

All CAR constructs used in this study are based on a second-generation CAR designed with 4-1BB as an intracellular costimulatory domain, and all expanded or quiescent CAR T cells were conditioned with human IL-7 and IL-15 post-EP or -LNP and prior to coculture with tumor cells. IL-7 increases mRNA translation in CAR T cells post-EP or LNP-mediated delivery by upregulating protein translation related pathways.^40^ Multiple previous reports have shown that preconditioning CAR T cells with IL-7 and IL-15 enhances the potency of CAR T cells and helps maintain naive-like and memory phenotypes of less differentiated T cells.^41^^,^^42^^,^^43^^,^^44^ Although both quiescent and expanded mRNA-based MVCAR demonstrated cytotoxic efficacy against GBM tumor cells, expanded MVCAR cells were superior both *in vitro* and *in vivo*. Notably, at low E:T ratios (2.4:1), expanded MVCAR outperformed monovalent CAR T cells as evidenced by enhanced cytokine secretions and cytotoxic potential. On the contrary, quiescent MVCAR failed to convey any additive cytotoxic potential to single-targeting CAR T cells *in vitro*. Quiescent LV-transduced CAR T cells have been reported to have more potent cytotoxicity at low E:T ratios in models of leukemia than traditionally expanded ones.^28^ Our data suggest that requirements for optimal production of quiescent or expanded CAR T cells might be different in the context of solid tumors as compared with blood malignancies. Optimization of CAR domains and conditioning medium is critical for fine-tuning the efficacy and safety of quiescent mRNA-based CAR T cells targeting solid tumors, which would become lifesaving for patients at advanced stages of tumor progression.

Upon testing in an NTR-operated xenograft s.c. NSG resection model of GBM, expanded MVCAR (Figure 6) demonstrated better safety profile with little to no GvHD symptoms than quiescent ones (Figure S3). Notably, responder mice achieving CR following quiescent MVCAR treatment suffered GvHD-related complications. Moreover, while quiescent Mock EP cells had little to no cytotoxic efficacy *in vitro* at the E:T ratios evaluated, around 83% of mice receiving quiescent Mock EP cells underwent CR that triggered lethal GvHD-associated complications. The exact mechanism of anti-tumor cytotoxicity in quiescent Mock EP cells is yet to be determined. Given the locoregional delivery of CAR T cells, one possible mechanism could be attributed to CAR-independent and T cell-intrinsic cytotoxicity due to the abundance of sheer number of T cells as compared with remaining tumor cells post-maximal surgical resection, which by far exceeds the E:T ratios tested *in vitro*, and reminiscent with what is seen with TILs. Additional contributing factors include mismatched HLA haplotypes between donor T cells and U87-MG cells, although this is less likely to be the determining factor, since such remissions were redundant with T cells from different donors, each having distinct HLA haplotypes, and were less severe in same donor-expanded T cells (Figure S3). Additional studies are needed to investigate the exploitation of such cytotoxic capacity of quiescent unedited T cells. This might provide a new avenue of treatment for extremely ill patients who require instant medical intervention but have limited access to CAR-modified therapies.

There are multiple considerations for achieving CR using mRNA-based CAR T cells. A key determining factor includes the EOR during surgery, whereby maintaining minimal residual disease that mirrors clinical outcomes is essential. Other determining factors are related to mRNA delivery platforms. In our platform, both MVCAR#2_LNP and MVCAR#2_EP cells demonstrated potent antitumoral cytotoxicity in the xenograft mouse models, regardless of differences in rhChimera binding, CAR frequency, and MFI. This is most probably attributable to the durable and considerably stable CAR expression that lasted up to 5 days for MVCAR#2_EP (Figure S1E) and more than 3 days for MVCAR#2_LNP (Figure 5N), in accordance with previously documented findings.^31^ Our findings further suggest that achieving potent and long-lasting remissions relies on balancing CAR signal durability with appropriate dosing timelines based on the identified therapeutic window. Quality and purity of *in-vitro*-transcribed (IVT) mRNA and composition of LNP formulation have a profound impact on mRNA expression durability.^45^^,^^46^ While in our system it worked well using in-house IVT mRNA and commercially available LNP formulation kit, it should be optimized in studies using different IVT kits and LNP formulation compositions. Another determining factor is the affinity of single scFv to cognate targets, or rhChimera. Moderate affinity scFv-based CAR T cells tend to demonstrate better clinical outcomes associated with longer persistence in four solid tumor clinical trials as compared with the high-affinity counterparts (reviewed in Mao et al.^47^). The EphA2#1 CAR [2A4] clone is an affinity-matured clone of the humanized monoclonal antibody [4H5], used in EphA2#2 CAR. We used flow cytometry staining with a fixed concentration of rhEphA2 chimera to validate the difference in affinity between 2A4 and 4H5 (Figure S5F). As anticipated, 4H5 scFv-containing triple-targeting MVCAR (MVCAR#2) had superior cytotoxicity *in vivo* as compared with those containing 2A4 scFv (MVCAR#1). Signaling interaction between tumor-associated antigens on tumor cells should also be considered. For instance, in a previous study, a cooperation was unfolded between two GBM prominent targets, IL13Rα2 and EGFRvIII, in promoting GBM progression.^48^ Hence, the choice of target combinations as well as scFv combinations should be optimized preclinically for achieving robust and enduring remission rates using mRNA-based MVCAR.

Relative to virally engineered CAR T cells, mRNA-based CAR T cells confer multiple distinct advantages that may enhance their clinical applicability and manufacturing scalability. For instance, mRNA-based CAR T cells (1) enable high and consistent CAR expression efficiencies, (2) eliminate the need of viral producer cells, (3) allow cost-effective and fast manufacturing that is amenable for multiplexing and automation, and (4) are less-restrained with regulatory restrictions.^24^ Although mRNA-based CAR T cells also exhibit an improved safety profile due to transient CAR expression—even in the event of off-target toxicity—the simultaneous targeting of multiple tumor antigens may increase the risk of unintended toxicities on healthy tissues. Hence, thorough analysis of target combinations and possible toxicities should be considered when developing mRNA-based MVCAR. Additionally, the cost and scalability of multi-targeting CAR T cell manufacturing as in MVCAR would vary significantly depending on (1) the routes of delivery (for example, EP versus the need for mRNA-LNP encapsulation and delivery), (2) limitations of multiplexing multiple mRNA products during delivery, and (3) storage conditions of CAR-encoding mRNA (frozen IVT mRNA or LNP-encapsulated mRNA). While mRNA-based CAR T cell manufacturing might be cost-effective, more in-depth evaluation and optimization of scalable multiplex mRNA-based CAR T cell manufacturing workflows is recommended, considering the variables related to the route of delivery and LNP-associated costs.

Moving forward, leveraging mRNA-based MVCAR seems a promising strategy against resectable solid tumors when combined with maximal surgical resection. However, locoregional delivery of multiple doses will be needed as inferred from our orthotopic model. Thus, optimization of locoregional delivery techniques and tools would help maximize MVCAR efficacy and infiltration into the TME. Other additional strategies that could be employed to further enhance the efficacy of our platform and improve the translational applicability of mRNA-based MVCAR include discovery and optimization of novel scFv constructs with optimal target affinities as well as *in silico* optimization of target combinations leveraging AI-assisted tools. Moreover, combining our platform with other immunotherapies used against solid tumors such as checkpoint inhibitors, T cell engagers, as well as novel adjuvant immunotherapies could further improve the translational potential and efficacy of mRNA-based MVCAR against solid tumors.

### Summary

mRNA-based CAR T cell models targeting GBM present a promising and innovative area of research, with advantages in terms of safety and flexibility. Our proposed unique three-pronged model offers a feasible and easily translational platform that enables proper preclinical evaluation of mRNA-based CAR T cells and subsequent development of rigorous clinical trials against GBM and other resectable solid tumors. With the feasibility of use of LNP-based or EP-based delivery, manufacturing of mRNA-based CAR T cells can be automated and easily performed at healthcare facilities and treatment centers. This reduces the cost and turn-around times for manufacturing and delivery, which is crucial for GBM patients, whose condition deteriorates quickly with tumor progression.

## Materials and methods

### Plasmids

The DNA sequences of scFv used in this study including anti-HER2 4D5,^49^ anti-IL13Ra2 Hu08,^20^ anti-EphA2 2A4^50^ and 4H5,^11^ anti-EGFR C225,^51^ and anti-CD19 FMC63^52^ were cloned into the mRNA transcription vector, pDA, containing a CAR-encoding backbone, including the CD8α leader sequence, a portion of the CD8α extracellular domain and transmembrane domain, 4-1BB, and CD3zeta endodomains (scFv-BBz) to generate pDA-CAR-BBz. For LV-based CAR T cells, 2A4-BBz was cloned into pTRPE vector to generate pTRPE-2A4-BBz, followed by LV particle purification and transduction into activated T cells as described previously.^52^ pTRPE-HER2 was constructed by Genscript, and LV particles were used for the generation of U87_HER2+ cells.

### *In vitro* transcription of 5′ capped poly(A)-tailed mRNA

A linearized DNA template was first prepared for each pDA-CAR-BBz plasmid by overnight incubation with restriction enzymes at 37°C followed by puri fication using a QIAquick PCR purification kit (QIAGEN). Later, mRNA was *in vitro* transcribed from the linearized DNA template using T7 mScript standard mRNA production system v.2 (CELLSCRIPT) as per the manufacturer’s manual. The post-transcriptional capping was performed by incubating uncapped IVT RNA with ScriptCap 2′-O-methyltransferase and ScriptCap capping enzyme for 1 h at 37°C. Subsequently, 5′ capped IVT RNA was incubated with A-plus poly(A) polymerase to produce ∼150 b long 3′ poly(A) tails.

The 5′ capped poly(A)-tailed IVT mRNA was purified using RNeasy mini kit (QIAGEN), quantified by NanoDrop One Spectrophotometer (Invitrogen), and verified by gel electrophoresis against an RNA marker. All mRNA samples were reconstituted to 1 or 2 μg/μL in molecular biology grade RNase-free and DNase-free water and stored at −80°C.

### U87-MG and primary T cell culture

U87-MG cells were obtained from the American Type Culture Collection (ATCC), cultured in a humidified incubator at 37°C and 5% CO_2_, and tested for mycoplasma. U87-MG and all derived cell lines were expanded in improved minimum essential medium (MEM) (Gibco) supplemented with 10% heat-inactivated fetal bovine serum (FBS) (Gibco), 1% GlutaMAX (Gibco), 1 mM sodium pyruvate (Gibco), 1% penicillin/streptomycin 50 U/mL (Gibco), and 1% HEPES (Gibco). None of the cells used in this study exceeded 10 passages of expansion.

Healthy donor primary T cells were obtained from the Human Immunology Core at the University of Pennsylvania and cultured in a humidified incubator at 37°C and 5% CO_2_. T cells were maintained in RPMI 1640 Media (Gibco) supplemented with 10% heat-inactivated FBS, 1% GlutaMAX, 1% penicillin/streptomycin 50 U/mL, and 1% HEPES (R10 media). For experiments in Figure 5, cells were maintained in ImmunoCult-XF medium (STEMCELL Technologies). Expanded T cells were obtained following stimulation with anti-human CD3/CD28 microbeads (Dynabeads, Gibco) at 1:1 ratio in T cell expansion medium supplemented with 5 ng/mL each of human IL-7 (PeproTech) and IL-15 (PeproTech). A LV encoding for a second-generation 2A4-BBz CAR was used for the generation of LV-transduced EphA2#1 CAR T cells (used in Figure 5).

### U87 cell line gene editing

U87-MG cell lines were transduced with an LV encoding human HER2 and sorted to purity. Sorted U87-HER2+ cells were then gene edited using CRISPR-Cas9 technology. Briefly, U87-HER2+ cells were washed with DPBS (Gibco) and resuspended in SE 4D-Nucleofetor X solution (Lonza). Individual sgRNA (IL13RA2-sgRNA: 5′-ATAGTGGATCCCGGATACTT-3ʹ; EGFR-sgRNA: 5′-AGTAACAAGCTCACGCAGTT-3ʹ; CSPG4-sgRNA: 5′-TCGGTCAGAGCCGTGGCCAC-3ʹ; EPHA2-sgRNA: 5′-GGTGATCTCATCGGGCGCAA-3′) (Synthego) were mixed with TrueCut Cas9 Protein v.2 (Invitrogen) and by incubated for 10 min at room temperature for RNP complex formation (Table S1). RNP mixed cell suspension was then moved to a Nucleofector cuvette and pulsed using the DS-126 program as per the 4D-Nucleofector manual. After nucleofection, U87_HER2+_target-KO cells were cultured for 7 days, then grown into single-cell clones (SCCs). Selected SCCs per cell line were verified with flow cytometry staining, TIDE analysis, and Synthego ICE analysis for 100% indel efficiency. Selected clones were then transduced with LV encoding Click Beetle Green (CBG) luciferase-P2A-GFP/NLS, CBG-P2A-BFP/NLS, or CBG-P2A-mKate2/NLS.

### RTCA xCELLigence eSight assays

Control or CAR T cells were cocultured with U87 target cells in E-plate view 96 (Agilent) or regular 96-well plates for 5 days at the indicated E:T ratios (noted in the article text and the figure legends). In some assays, Incucyte Cytotox Red Dye (Sartorius) was added to the cocultures to quantify target cell death in real time. Triton X-100 (1%) was added to target cells in replicate wells as a positive control for maximal cell lysis. All cocultures were run in duplicates or triplicates depending on the assay. Real-time normalized impedance and imaging analyses were recorded on the xCELLigence RTCA eSight machine (Agilent). At 120 h post-coculture, the experiment was terminated and data from normalized cell index (for impedance analysis) or fluorescent intensities (for imaging analysis) were exported using xCELLigence RTCA eSight software (Agilent) and used to calculate percent cytolysis.

### Flow-based cytotoxicity assay

Control T cells or CAR T cells were cocultured with U87 target cells in 96-well plates for 5 days at the indicated E:T ratios. At 120 h post-coculture, cells were transferred to a round-bottom 96-well plate and stained prior to running on LSRFortessa flow cytometry. Absolute live cell counts were obtained by normalizing to the number of precision count beads/well (BioLegend).

### Luciferase cytotoxicity assay

CBG luciferase-expressing U87 target cell lines were used in these assays. Control T cells or CAR T cells were cocultured with U87 target cells in 96-well plates at indicated E:T ratios for 48 h. Cells were then lysed in 1× reporter lysis buffer (Promega). Lysates were mixed with luciferin substrate (Promega) and luminescence was analyzed using a BioTek Synergy H4 Hybrid Multi-Mode Microplate Reader (Agilent). Specific lysis of each sample was then calculated. Triton X-100 (1%) was added to target cells in replicate wells as a positive control for maximal cell death.

### Cell proliferation assay

U87 cell lines were stained with CellTrace CFSE dye (Invitrogen) at 1/1,000 dilution prior to plating at 1e–4 cells/well, 4 replicate wells/cell line in a 96-well plate. Real-time proliferation analysis was performed by monitoring cell growth using the image-only module on the eSight machine (Agilent). Five days later, cells were stained with LIVE/DEAD Fixable Violet dye (Invitrogen) and analyzed on a BD LSRFortessa flow cytometer (BD Biosciences), where absolute live cell counts were obtained and normalized to precision count beads.

### GSC target expression profiling and cytotoxicity assays

Three patient-derived GSC lines (nos. 5077, 8977, and 8979) were a gift from the labs of Dr. Donald O’Rourke and Dr. Zev Binder, University of Pennsylvania. Cells from each GSC line were stained with antibodies targeting HER2, IL13Rα2, and EphA2 and analyzed on a BD LSRFortessa flow cytometer (BD Biosciences). For the cytotoxicity coculture assay, GSC cells were digested with Accutase, stained with CellTrace Yellow (CT-Y) according to manufacturer’s instructions, and seeded in 96-well plates at 1e–5/0.1 mL/well. CAR+ T cells in 0.1 mL were distributed to coculture plates at T cell:GSC ratios of 3:1, 1:1, 0.3:1, and 0.1:1 in triplicate for each CAR T+ condition and GSC line. Twenty-four or 96 h after coculture, cells were stained with a viability dye and anti-CD3 fluorophore antibody. Samples were analyzed on a BD LSRFortessa flow cytometer (BD Biosciences). Absolute live cell counts were obtained by normalizing to the number of precision count beads/sample (BioLegend).

### ELISA

For the comparison between expanded and quiescent T cells, Mock EP or CAR T cells were incubated with U87KOpool cells at an E:T ratio of 2.4 (CAR+ cells):1 in cytokine-free medium. Twenty-four hours post-coculture, supernatants were collected to assess human IL-2 or IFN-γ cytokine production. The cytokine measurements were performed using respective DuoSet ELISA kits (R&D Systems) according to the manufacturer’s instructions. For the assessment of IFN-γ secretion following coculture of U87 single target KO cells or U87KOpool cells with single-targeting CAR T cells in Figure S1, an E:T ratio of 10:1 was used.

### LNP formulation and verification

Microfluidic formulation of mRNA-LNPs was performed using a GenVoy-ILM T cell kit for mRNA, Ignite, and a NanoAssemblr_ Ignite device (Precision NanoSystems) as per the manufacturer’s guidelines, at a 2:1 aqueous to lipid ratio, and total flow rate of 12 mL/min. For triple-targeting MVCAR formulation, the total amount of RNA remained constant, and was divided by three for each mRNA. After formulation, the formulated mRNA-LNPs were diluted 30× with 1× dilution buffer and dialyzed through an Amicon ultracentrifugal filter, 30 kDa MWCO (Millipore) prior to reconstitution to original volume. Characterization of the diameter (z-average) and PDI of the formulated mRNA-LNPs was performed on a Zetasizer Nano ZS (Malvern Panalytical) using the following parameters: measurement type, size; material, protein; dispersant, DPBS; temperature, 25°C; equilibration time, 120 s; cell type, ZEN0040; measurement angle, 173° backscatter; number of runs, 12; run duration, 10 s; number of measurements, 3 with no delay.^31^ The encapsulated mRNA was quantified using the Quant-iT RiboGreen Assay (Invitrogen) as per the protocol provided by Precision NanoSystems. Briefly, to determine the concentration of free and total mRNA for calculations of encapsulated mRNA and encapsulation efficiency, mRNA-LNPs were incubated in 1× TE buffer for 10 min at 37°C in the absence or presence of Triton X-100 (to dissolve the lipid layer), followed by addition of 100 μL of the Quant-iT RiboGreen RNA Reagent, diluted 1:100 in 1× TE buffer. Fluorescence was measured at λ_ex/em_ = 485/528 nm and read height 8 mm on a BioTek Synergy H4 Hybrid Multi-Mode Microplate Reader (Agilent). mRNA encapsulation concentration and efficiency were calculated against values of an RNA standard curve.

### EP-mediated delivery of mRNA into T cells

For quiescent T cells, freshly isolated or thawed T cells were washed with 1× DPBS prior to resuspension in Opti-MEM medium (Gibco). For monovalent single-targeting CAR T cell generation, mRNA was added at 1 μg per 1e6 T cells for each of 4D5-BBz, C225-BBz, 228.15S-BBz, and Hu08-BBz or at 1.1 μg per 1e6 T cells for 2A4-BBz or 4H5-BBz, and EP was performed on an ECM 830 square-wave electroporation system (BTX) using the following settings: voltage, 500 V; pulse length, 1 ms; number of pulses, 1. For MVCAR#1 T cell generation, the mRNA mix used was optimized as follows: (1:1:1.1 μg of 4D5-BBz:Hu08-BBz:2A4-BBz mRNA per 1e6 T cells) and for MVCAR#2 cells: (1:1:1.1 μg of 4D5-BBz:Hu08-BBz:4H5-BBz mRNA per 1e6 T cells). Following expansion, EP-ed cells were moved to flasks containing R10 medium-supplemented cells with 5 ng/mL of each of human IL-7 and IL-15. For expanded CAR T cell generation, T cells were expanded with either R10 or ImmunoCult expansion medium supplemented with 5 ng/mL of each of human IL-7 and IL-15 using CD3/CD28 microbeads for 4 days. Subsequently, cells were washed with 1× DPBS prior to resuspension in OPTIMEM medium and EP on an ECM 830 system using the #918 protocol. mRNA concentrations and combinations were the same as those used for quiescent T cells. Following EP, cells were further incubated in cytokine supplemented medium. Sixteen to 24 h post incubation, CAR T cells were counted, stained for CAR expression, and used in functional assays or directly injected into mice.

### LNP-mediated delivery of mRNA into T cells

T cells were expanded in ImmunoCult-XF medium supplemented with 5 ng/mL each of human IL-7 and IL-15 (ImmunoCult T cell expansion medium) using CD3/CD28 microbeads. On day 3, T cells were resuspended at 0.5 e6/mL in T cell expansion medium in the presence of 1 mg/mL ApoE4 (GenVoy-ILM T cell kit for mRNA from Precision NanoSystems). mRNA-LNP was added at 1 μg of encapsulated mRNA per 1e6 expanded T cells, and cells were incubated at 37°C and 5% CO_2_. On day 4, CAR T cells were analyzed for CAR staining by running on an LSRFortessa flow cytometer and used in functional *in vitro* assays or for injection in mice.

### Flow cytometry staining

CAR expression and surface markers of viable T cells and U87 cells were analyzed on a BD LSRFortessa flow cytometer. A list of the antibodies used in staining is supplemented in Table S2. Cells were washed with 2% FBS-supplemented DPBS (FACS) buffer. Subsequently, cells were stained with fluorophore-conjugated antibodies for 25 min at 4°C. To quantify the cells, precision count beads were used as per the manufacturer’s guidelines. Gating was based on singlet gating, exclusion of dead cells using LIVE/DEAD dyes from Invitrogen, then forward versus side scatter gating to identify the population of interest. CAR staining was performed using biotin-conjugated goat F(ab′)_2_ anti-human IgG (H + L) (Jackson ImmunoResearch) followed by staining with fluorophore-conjugated streptavidin. Sample results were analyzed using FlowJo v.10 software (BD Biosciences).

CAR T cell staining with rhChimera (obtained from R&D Systems) was performed by first staining with rhEphA2-HisTag (7 μg/mL) followed by staining with anti-His Tag-Pacific Blue (BioLegend). Cells were then washed twice to remove any unbound anti-His Tag antibody. Then, a mastermix of rhHer2-AF488 (9 μg/mL) and rhIL13Rα2-AF647 (2 μg/mL) was prepared and used for staining the cells. Viability was determined by gating on a LIVE/DEAD Aqua-negative population.

### Xenograft s.c. resection model

All protocols for aseptic survival surgery and handling of mice were approved by the Institutional Animal Care and Use Committee at the University of Pennsylvania, in accordance with Federal and Institutional Animal Care and Use Committee requirements. NOD.Cg-Prkdc^scid^ Il2rg^tm1Wjl^/SzJ (NSG) mice aged 6–10 weeks were originally obtained from the Jackson Laboratory and bred by the Stem Cell and Xenograft Core (SCXC) at the University of Pennsylvania. Mice were inoculated with U87KOpool cells (U87-HER2+_EphA2KO_CBG+, U87_HER2+_IL13Rα2KO_CBG+, and U87_HER2null-CBG+ cells in equal ratios) and were monitored once or twice a week for tumor growth and body weight.

Two days prior to aseptic survival surgery, mice were shaved at the tumor-bearing side and imaged using an IVIS SpectrumCT (PerkinElmer). Surgery was performed when the tumor was palpable and tumor BLI measurements reached an average total flux between 10^9^ and 10^10^ photon/s (p/s). Mice were randomized into treatment cohorts such that the average total flux was equal in each cohort. Animals either underwent partial (STR) or near-total (NTR) surgical excision that maintains minimal residual disease, with details of excision specified for each mouse model in the relevant figures. Surgery was performed with adaptation to previously published protocols.^35^^,^^53^^,^^54^ In short, a 2 × 1 cm incision was made around the tumor site, keeping extra skin area to easily close the incision once done. Approximately 70% (for STR) or 95% (for NTR) of the capsule containing the tumor was carefully removed from the skin side, making sure the resection cavity remained intact. The remaining tumor was kept attached to the skin subcutaneously to maintain existing vasculature. Sterile DPBS or T cells suspended in DPBS were applied within the subcutaneous skin layer at the resection site. To close the surgical site, the skin sides of the incision were pulled together with bent forceps, and Autoclip 9 mm wound clips (Becton Dickinson) were applied to close the incision. Mice were monitored for 72 h for signs of redness, inflammation, or wound dehiscence. Different healthy donor T cells were used in each animal model.

### Peripheral blood staining

Retro-orbital bleeding or cardiac puncture were used to collect peripheral blood from NSG mice, followed by staining inside TruCount tubes (BD Biosciences) as per the manufacturer’s guidelines. Samples were acquired on LSRFortessa flow cytometer and analyzed using FlowJo v.10 software. Cell numbers of CD4 or CD8 T cells were calculated per μL of blood, and the percentage distribution of Tim-3 and/or PD-1 positive cells was analyzed.

### Splenocyte and tumor cell isolation and staining

Splenocytes were collected by squeezing out of the capsule and straining through a 70 μm strainer. Disaggregated splenocytes were then treated with ammonium-chloride-potassium (ACK) Lysing Buffer (Quality Biological) to lyse red blood cells, prior to staining an equal number of cells per mouse with fluorophore-conjugated antibodies and LIVE/DEAD stain for exclusion of dead cells.^55^ Precision count beads were added to normalize cell counts. Samples were acquired on an LSRFortessa flow cytometer and analyzed using FlowJo v.10 software.

Tumors were collected from mice, and U87-sourced tumor cells were homogenized using a modified protocol from Baker et al.^56^ Briefly, the tumor bulk was triturated by cutting and smashing with the flat end of the syringe plunger. Triturated tumor matter was then homogenized by incubating in a mix of 1 mg/mL Collagenase IV (Worthington Biochemical) and 20 U/mL DNase I (Thermo Scientific) at 37°C for 30 min. Cells were then strained through a 70 μm strainer, treated with ACK buffer to lyse red blood cells, and resuspended in FACS buffer. Antibodies were added to stain for T cells or target antigens as described in the figure legends. Precision count beads were added to normalize cell counts. Samples were acquired on an LSRFortessa flow cytometer and analyzed using FlowJo v.10 software.

### Orthotopic glioma mouse model

Orthotopic glioma tumors were established by stereotactic injection of 25,000 patient-derived Ge518 glioma cells into the striatum. Mice were anesthetized with a general anesthetic and received subcutaneous buprenorphine for perioperative analgesia, along with local infiltration of lidocaine at the scalp incision site. Animals were positioned in a stereotaxic frame in the prone position and maintained on a thermostatically controlled heating pad throughout the procedure. A small midline scalp incision was performed, and a burr hole was drilled at stereotaxic coordinates relative to bregma: anteroposterior +0.5 mm, mediolateral +2.0 mm, dorsoventral −3.0 mm. Tumor cells were injected in a volume of 3 μL using a Hamilton syringe inserted into the striatum after a 2-min dwell time to reduce reflux and the needle was slowly withdrawn. The skin was closed using surgical glue, and mice were allowed to recover on a thermostatically controlled blanket, with *ad libitum* access to food and water under a 12 h light/dark cycle. Tumor growth was monitored by using BLI with an IVIS Lumina S5 system (PerkinElmer), starting from day 9 post-injection for randomization and performed at regular intervals thereafter.

### Intracranial tumor resection model

Ten days post-tumor implantation, animals underwent surgery under general anesthesia and received subcutaneous buprenorphine for analgesia, along with local infiltration of lidocaine at the incision site. Mice were positioned prone on a stereotaxic frame and maintained on a thermostatically controlled heating pad throughout the procedure. A paramedian skin incision (15–20 mm in length), extending the initial injection site, was performed. Craniectomy was carried out using a 3 mm trephine centered on the pre-existing cranial opening. Following cortico-dural coagulation, macroscopically visible tumor tissue was removed by aspiration using a diaphragm vacuum pump (VACUSON 60, Nouvag). Hemostasis was achieved using surgical hemostatic compresses and irrigation with sterile physiological saline. The resection cavity was then filled with Encapgel (33% in PBS; 922412-1EA, Sigma-Aldrich), either alone or loaded with CAR T cells (10^6^ CAR-positive T cells in 5 μL). The cranial window was sealed, and the skin was closed using surgical glue. Postoperative care included placement on a thermostatically controlled blanket, with *ad libitum* access to food and water under a 12 h light/dark cycle.

### Data presentation and statistical analysis

GraphPad Prism v.10 was used for statistical analysis and graphical plotting of data. The number of samples and state of statistical significance is plotted on figures and mentioned in the figure legends. Grouped data are plotted as mean of donor samples or individual replicates per analyzed groups plus or minus standard deviation of the mean (SD). Unpaired or paired two-tail t tests or nonparametric Mann-Whitney U tests were used to analyze data between two groups. Statistical analysis of the means of three or more groups was performed using ordinary one-way analysis of variance (ANOVA) or nonparametric Kruskal-Wallis ANOVA followed by appropriate post hoc comparison analysis. Multivariate grouped datasets were analyzed by two-way ANOVA or mixed-effect multiple comparison analysis (in the case of missing data), followed by appropriate post hoc comparison analysis. Survival curves were plotted using the Kaplan-Meier method based on the log rank Mantel-Cox test. For all data analyzed, ns denotes not significant, ∗*p* < 0.05, ∗∗*p* < 0.01, ∗∗∗*p* < 0.001, and ∗∗∗∗*p* < 0.0001. Licensed BioRender images were used where necessary (www.biorender.com).

## Data availability

The data that support the key findings of this study are available within the article and supplemental material.

## Acknowledgments

The authors thank David Degaramo and Fang Liu of the Posey lab for technical support with this project. Additionally, the authors also thank Max Eldabbas, Emileigh Maddox, Tanishk Sinha, and Jiayi Shu of the Human Immunology Core (HIC) at the Perelman School of Medicine at the University of Pennsylvania for providing normal donor T cells. The HIC is supported in part by 10.13039/100000002NIH P30 AI045008 and P30 CA016520. The authors thank Anthony Secreto and Joshua Glover of the Stem Cell & Xenograft Core (SCXC) at the Perelman School of Medicine at the University of Pennsylvania for their support and training on NSG animal handling and survival surgery. The authors also thank the lab of Dr. Jacob Brenner, especially Aparajeeta Majumder, for providing access and support in conducting zetasizer measurements. The authors also thank the lab of Dr. Donald O’Rourke and Dr. Zev Binder, especially Logan Zhang, for providing patient-derived GSCs. The authors would also like to thank the reviewers for their insightful inputs and suggestions. This work was supported by funding from Penn Medicine’s GBM Translational Center of Excellence, the 10.13039/100000002National Institutes of Health U54CA244711, the Department of Veteran Affairs Office of Research and Development I01BX006247 (to A.D.P), and the 10.13039/501100017035ISREC Foundation (to D.M.). Some diagrams were created using BioRender.

## Author contributions

Conceptualization, O.K.D., D.M., and A.D.P.; data curation, O.K.D.; formal analysis, O.K.D.; funding acquisition, D.M. and A.D.P.; investigation and methodology, O.K.D., M.P., D.M.B., and S.K.B.; visualization, O.K.D.; resources, O.K.D., D.M., and A.D.P.; writing – original draft, O.K.D.; writing – review & editing, O.K.D., D.M., and A.D.P.

## Declaration of interests

O.K.D. is an inventor of patent related to CAR T cell therapy, filed by the University of Pennsylvania. D.M. is an inventor of patents related to CAR T cell therapy, filed by the University of Pennsylvania, the Istituto Oncologico della Svizzera Italiana (IOSI), and the University of Geneva, and is a consultant for Limula Therapeutics and MPC Therapeutics. D.M. is the scientific cofounder of Cellula Therapeutics SA. A.D.P. is an inventor of patents related to CAR T cell therapy, filed by the University of Pennsylvania and the University of Geneva, and is a consultant for Astellas Pharma and ImmunoACT.

## Supplemental information

Document S1. Figures S1–S7 and Tables S1 and S2

Document S2. Article plus supplemental information
