# Supplementary material for: Preclinical efficacy of multi-targeting mRNA-based CAR T cell therapy in resection models of glioblastoma
Source: Mol Ther Nucleic Acids. 2025 Aug 11;36(3):102676. doi: 10.1016/j.omtn.2025.102676 (PMC12397939; doi:10.1016/j.omtn.2025.102676)
Supplement: Document S1. Figures S1–S7 and Tables S1 and S2 [file mmc1.pdf]

**OMTN, Volume *36***

## Supplemental information

# Preclinical efficacy of multi-targeting mRNA-based CAR T cell therapy in resection models of glioblastoma

**Oula K. Dagher, Martin Pedard, Darel Martinez Bedoya, Shawna K. Brookens, Denis Migliorini, and Avery D. Posey Jr.**

**Supplemental Material**

**Table S1.** Table shows sequences of sgRNA, PCR primers, and sequencing primers used for knockout of target genes from U87_HER2+ cells by CRISPR/Cas9 and subsequent analysis of gene editing efficiency in genomic DNA extracts.

| Target | sgRNA Sequence | PCR Forward Primers | PCR Reverse Primers | Seq Primers |
|---|---|---|---|---|
| IL13RA2-sgRNA | 5'ATAGTGGATCC CGGATACTT3'_F | 5'TATGTGGTCTTTTGT GTCTGGC3' | 5'GAAAAGGTCACAGG TCAGTAGC3' | 5'AATTGGTGCACATTTG GAAGCC3'_F |
| EPHA2-sgRNA | 5'GGTGATCTCAT CGGGCGCAA3'_R | 5'GAAAGACCAGGGGC CAGATTCC3' | 5'GCATCCTCCACCTT CTCGTAGC3' | 5'GCTCGGGGCACTTCT TGTAGTA3'_R |
| EGFR-sgRNA | 5'AGTAACAAGCT CACGCAGTT3'_F | 5'TGCCTACTGGAGCT CTTACAGG3' | 5'TTCAAGTGGAATTCT GCCCAGG3' | 5'TGGGCAGGAATGGGT GAGTC3'_F |
| CSPG4-sgRNA | 5'TCGGTCAGAGC CGTGGCCAC3'_R | 5'GGTGCCAGGACCAA GAAGATGC3' | 5'TTGTGGCGGGAAAC ATGGTTGG3' | 5'AAGCTAGAGAAGGAA TCCCACC3'_R |

**Table S2.** List of antibodies used for flow cytometry staining

| Human Targets / Stains | Clone | Conjugate / Fluorophore | Catalogue Number | Vendor |
|---|---|---|---|---|
| CD3 | OKT3 | BV605 | 317322 | BioLegend |
| CD4 | SK3 | BUV395 | 563550 | BD Biosciences |
| CD45 | 5B1 | APC-Vio® 770 | 130-113-115 | Miltenyi Biotech |
| CD8 | SK3 | BUV805 | 612890 | BD Biosciences |
| Tim-3 | F38-2E2 | PE/Cyanine5 | 345052 | BioLegend |
| PD-1 | EH12.1 | BB700 | 566460 | BioLegend |
| EphA2 | 371805 | BV711 | BDB748149 | BD Biosciences |
| HER2 | 24D2 | APC-Fire750 | 324422 | BioLegend |
| IL13Rα2 | SHM38 | PE | 354404 | BioLegend |
| goat anti-human $F(ab)'_2$ | Polyclonal | Biotin | 109-066-006 | Jackson Immuno Research |

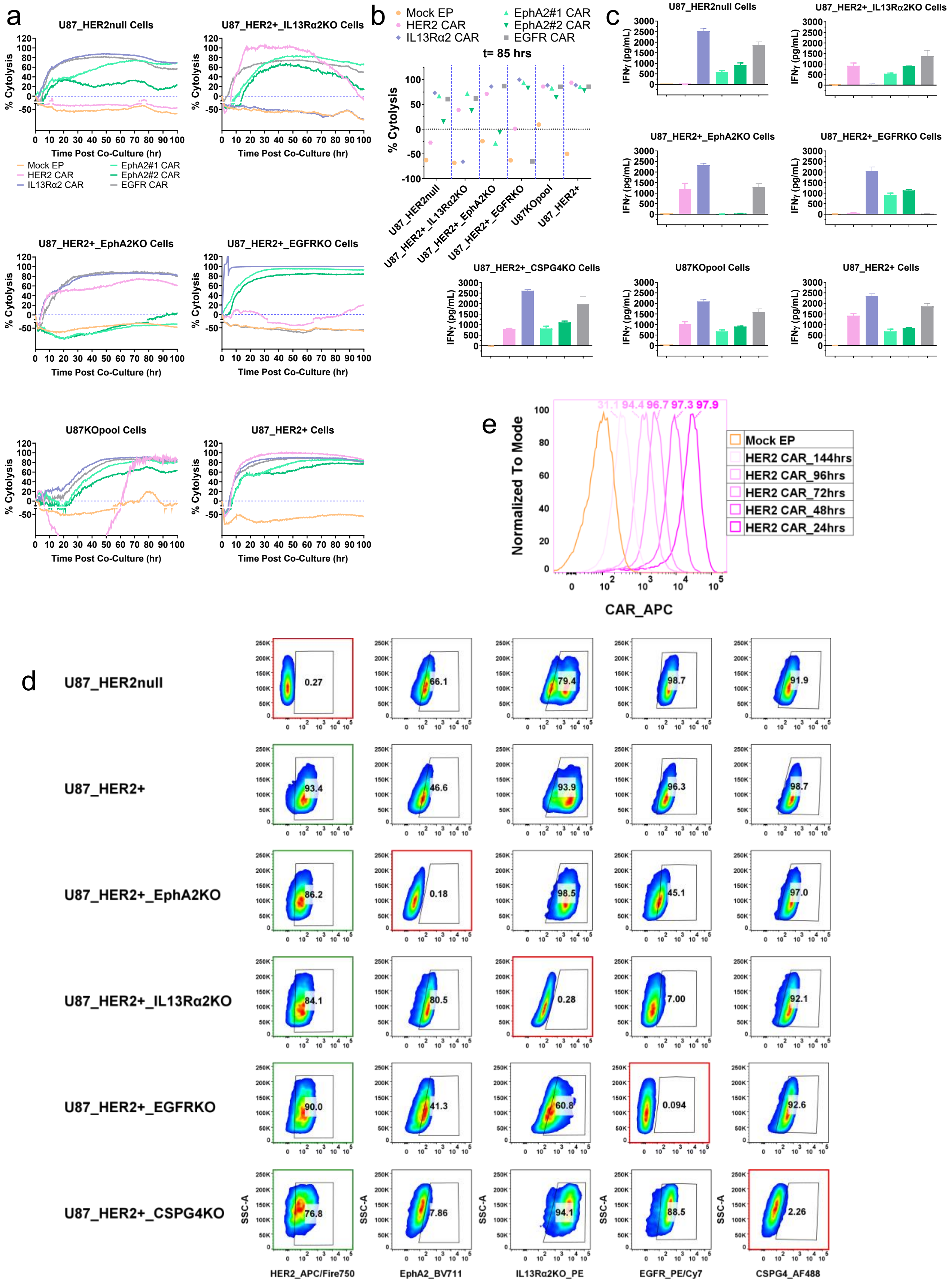
a
U87_HER2null Cells
U87_HER2+_IL13Rα2KO Cells
U87_HER2+_EphA2KO Cells
U87_HER2+_EGFRKO Cells
U87KOpool Cells
U87_HER2+ Cells
% Cytolysis
Time Post Co-Culture (hr)
Mock EP
HER2 CAR
IL13Rα2 CAR
EphA2#1 CAR
EphA2#2 CAR
EGFR CAR
b
t= 85 hrs
U87_HER2null
U87_HER2+_IL13Rα2KO
U87_HER2+_EphA2KO
U87_HER2+_EGFRKO
U87KOpool
U87_HER2+
c
U87_HER2+_CSPG4KO Cells
IFNγ (pg/mL)
e
Normalized To Mode
CAR_APC
31.1 94.4 96.7 97.3 97.9
HER2 CAR_144hrs
HER2 CAR_96hrs
HER2 CAR_72hrs
HER2 CAR_48hrs
HER2 CAR_24hrs
d
U87_HER2null
U87_HER2+
U87_HER2+_EphA2KO
U87_HER2+_IL13Rα2KO
U87_HER2+_EGFRKO
U87_HER2+_CSPG4KO
SSC-A
HER2_APC/Fire750
EphA2_BV711
IL13Rα2KO_PE
EGFR_PE/Cy7
CSPG4_AF488

**Fig. S1. Functional analysis and phenotypic verification of single-targeting mRNA-based CAR T cells and U87KO cell lines. (a-c):** Functional analysis of single-targeting mRNA-based CAR T cells on U87KOpool cells. Efficient Antigen (Ag)-dependent and bystander killing, respectively, of pooled Ag (+) and Ag (-) U87 tumor cells (in U87KOpool) by single-targeting CAR T cells. Single-target KO or HER2+ U87 cells were used as controls to validate individual CAR-BBz cell specific lysis of target tumor cells. Percent cytolysis was calculated based on normalized cell index obtained from RTCA eSight software (**a-b**, n=2 biological replicates sourced from one healthy donor and plotted as mean % cytolysis). (**c**): Target-binding induced secretion of IFNγ from CAR T cells post-coculture with indicated U87 target cells, as measured by ELISA ( n=2 biological replicates sourced from one healthy donor and plotted as mean ± SD). (**d**): Phenotypic verification of U87 edited cell lines. Single cell clones of edited U87 cells were flow-stained with a mastermix of antibodies against all five targets for phenotypic verification of gene edits performed. (**e):** Representative longitudinal assessment of CAR expression in mRNA-based CAR T cells. mRNA-based HER2 CAR T cells prepared from healthy donor T cells were monitored over 6 days by flow staining for CAR expression.

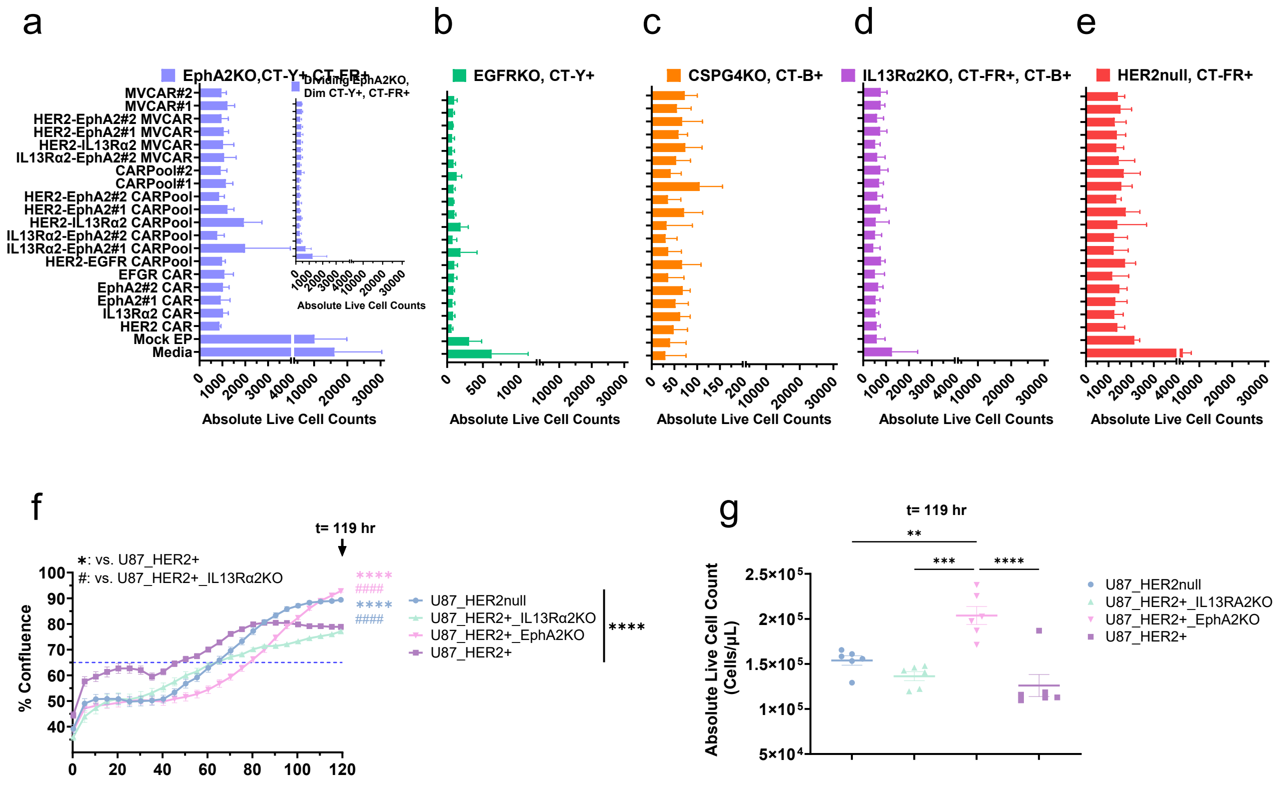


**Fig. S2. Phenotypic proliferation analysis of U87KO cell lines. (a-e):** Flow-based killing assay of dual or triple targeting CARPool or MVCAR against U87KOpool cells. Distinct U87_KO cells were stained with Cell-Trace® (CT) yellow (CT-Y: EGFRKO cells), blue (CT-B: CSPG4KO cells), far-red (CT-FR: HER2null cells), yellow + far red(CT-Y + CT-FR: EphA2KO cells), or blue + far red(CT-B + CT-FR: IL13Rα2KO cells) prior to pooling in equal ratios and coculture with effector cells. All U87_KO subgroups were seen in remnant live tumor cells within all treatment groups. The differences in percent population distribution for each of HER2null, EGFRKO, and EphA2KO cells were statistically significant across all CAR-BBz treatment groups as compared to targets only (Media) (statistical symbols not shown for better clarity of bar graphs. N= 2 donors, 2 replicates each, per assay. **(f-g):** Distinct proliferation capacity of U87 edited cells. Selected U87 edited cell lines were monitored for real time proliferation by RTCA eSight imaging module (n=6 replicates; one-way ANOVA followed by Tukey comparison analysis). The percentage mean brightfield (BF) confluence change

from $t_0$ was calculated **(f)**. Around 5 days post coculture, cells were stained for viability and ran on flow cytometry, where absolute counts were obtained **(g).**

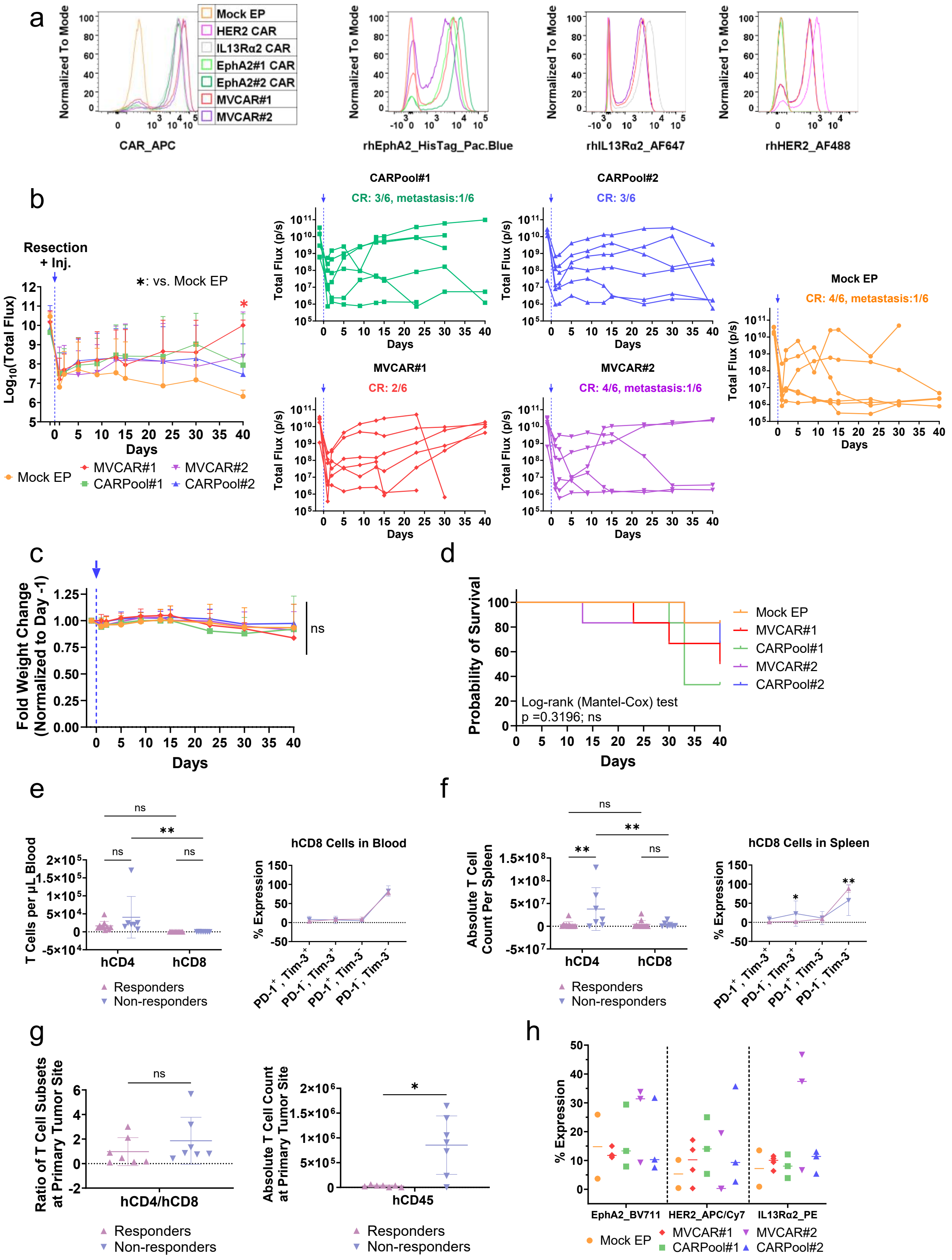
a
Mock EP
HER2 CAR
IL13Rα2 CAR
EphA2#1 CAR
EphA2#2 CAR
MVCAR#1
MVCAR#2
Normalized To Mode
CAR_APC
rhEphA2_HisTag_Pac.Blue
rhIL13Rα2_AF647
rhHER2_AF488
b
Resection + Inj.
*: vs. Mock EP
Log10(Total Flux)
Days
Mock EP
MVCAR#1
MVCAR#2
CARPool#1
CARPool#2
CARPool#1
CR: 3/6, metastasis:1/6
CARPool#2
CR: 3/6
Mock EP
CR: 4/6, metastasis:1/6
MVCAR#1
CR: 2/6
MVCAR#2
CR: 4/6, metastasis:1/6
Total Flux (p/s)
c
Fold Weight Change (Normalized to Day -1)
ns
Days
d
Probability of Survival
Log-rank (Mantel-Cox) test
p =0.3196; ns
Days
e
T Cells per µL Blood
hCD4
hCD8
Responders
Non-responders
hCD8 Cells in Blood
% Expression
PD-1+, Tim-3+
PD-1-, Tim-3+
PD-1+, Tim-3-
PD-1-, Tim-3-
f
Absolute T Cell Count Per Spleen
hCD4
hCD8
Responders
Non-responders
hCD8 Cells in Spleen
% Expression
g
Ratio of T Cell Subsets at Primary Tumor Site
hCD4/hCD8
Absolute T Cell Count at Primary Tumor Site
hCD45
Responders
Non-responders
h
% Expression
EphA2_BV711
HER2_APC/Cy7
IL13Rα2_PE
Mock EP
MVCAR#1
MVCAR#2
CARPool#1
CARPool#2

**Fig. S3. Comparison between quiescent Mock EP or mRNA-based multi-targeting CAR T cell cytotoxicity in NTR-operated mice.** Mice bearing U87KOpool-CBG+ cells in the left flank were subjected, on day 0, to NTR-operation and locoregional Mock EP, CARPool, or MVCAR injections, all sourced from healthy donor #ND637. (**a**): CAR staining and rhChimera staining of T cells used for injections. Graphs in (**b**) depict mean ± SD of log-transformed total flux (mixed-effect comparison analysis, post hoc Tukey, n=6 mice/group) as well as individual plots of longitudinal total flux per treatment group. No significant differences were observed between quiescent Mock EP or CAR T cell treatment groups in terms of weight change (**c,** mixed effect multiple comparison analysis, post hoc Tukey) or probabilities of survival, plotted as Kaplan-Meier survival analysis (**d**, logrank (Mantel-Cox) test, p= 0.3196, ns). Samples collected from blood, spleen, and primary tumor site during sacrifice were analyzed by flow staining. Graphs (**e-g**) show comparison analyses of T cell subsets in the blood, spleen, and tumor site of responders versus non-responders within the pooled CAR-cells treated mice. (**e-f**): Mixed effect multiple comparison analysis, based on uncorrected Fisher's LSD or Tukey analysis. (**g**): Mann-Whitney U test. (**h**): Staining of remnant tumor cells at primary tumor sites with antibodies against HER2, EphA2, and IL13Rα2 showed no significant differences between treatment groups (mixed effects comparison analysis, post hoc Tukey, ns).

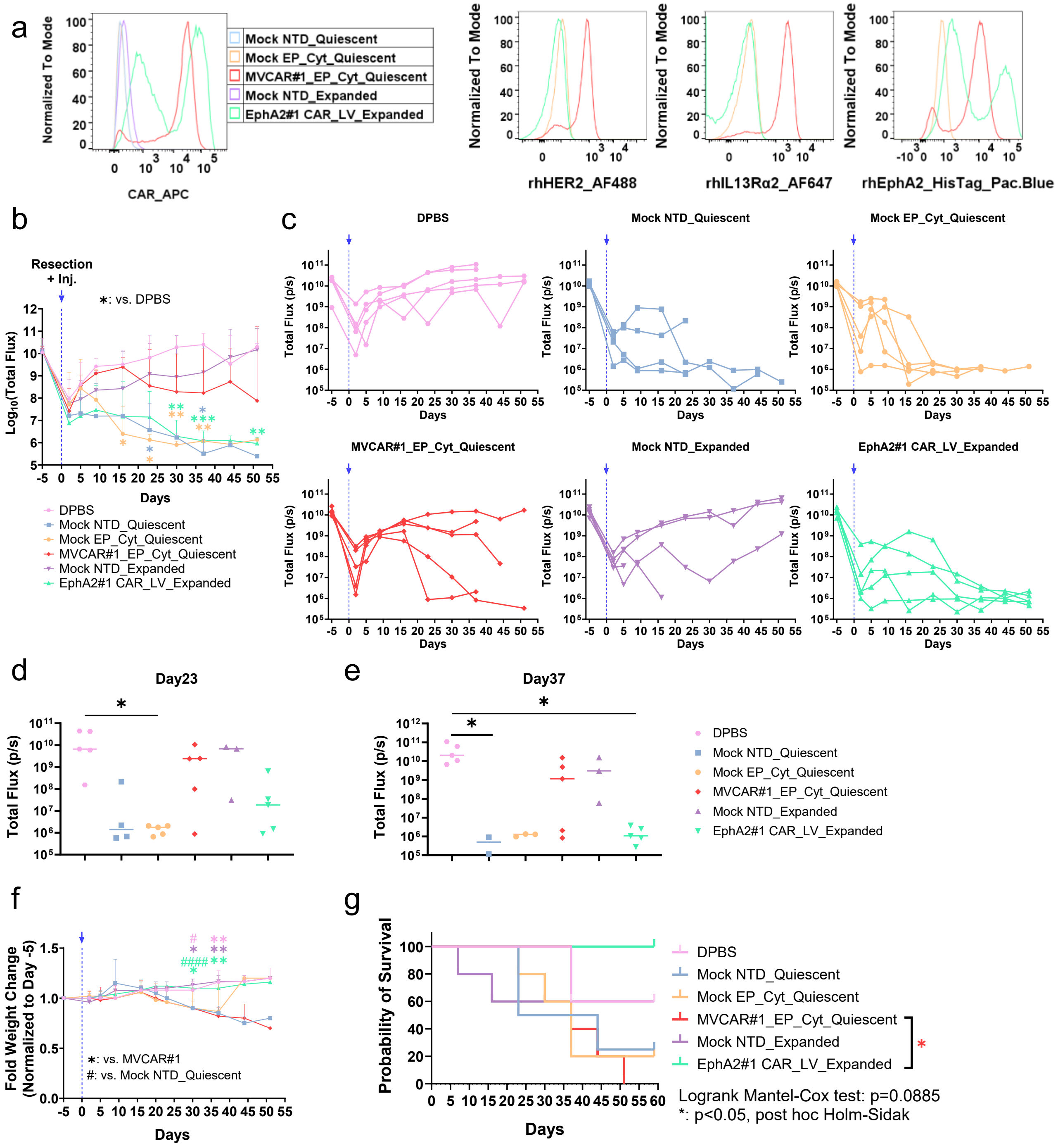


**Fig. S4. Influence of expansion on the cytotoxicity and safety of unedited T cells.** Mice bearing U87KOpool-CBG+ cells in the left flank were subjected, on day 0, to NTR-operation and locoregional injections of DPBS, Mock NTD_Quiescent T cells, Mock EP_Cyt_Quiescent T cells conditioned in IL-7/IL-15,

quiescent mRNA-based MVCAR#1_EP cells conditioned in IL-7/IL-15 (MVCAR#1_EP_Cyt_Quiescent), Mock NTD T cells expanded in IL-7/IL-15 (Mock NTD_Expanded), or LV-transduced EphA2#1 CAR T cells expanded in IL-7/IL-15 (EphA2#1 CAR_LV_ Expanded). All cells were sourced from healthy donor #TMP518. Flow histograms in (**a**) show CAR staining with anti-F(ab')$_2$_APC or rhChimera of T cells used for injections. (**b**) mean ± SD of log-transformed total flux of all groups (mixed-effect comparison analysis, post hoc Tukey pairwise analysis, n=5 mice/group). (**c**) Individual total flux data per group. Graphs in (**d-e**) show the differences in mean ± SD total flux at days 23 (**d**) and 37 (**e**) post-(NTR plus injections of T cells); Kruskal-Wallis ANOVA, post hoc Dunn's multiple comparison analysis. (**f**): Mean ± SD of the longitudinal fold change in body weight normalized to day -5 before treatment (mixed effects comparison analysis, post hoc Tukey). (**g**): Kaplan-Meier survival analysis by log-rank Mantel-Cox, post hoc Holm-Sidak. Although the overall logrank test comparing all groups was not significant (p=0.0885), a Holm-Sidak post hoc pairwise comparison revealed a significant difference between MVCAR#1_EP_Cyt_Quiescent and EphA2#1 CAR_LV_ Expanded T cells.

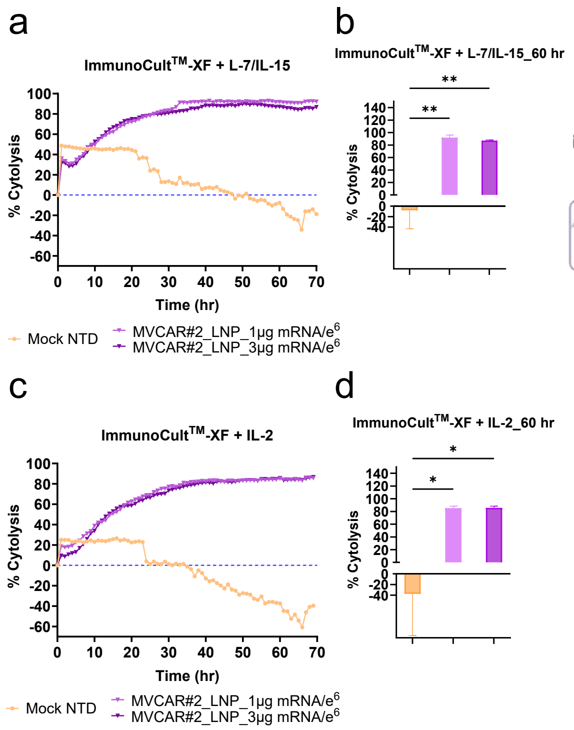


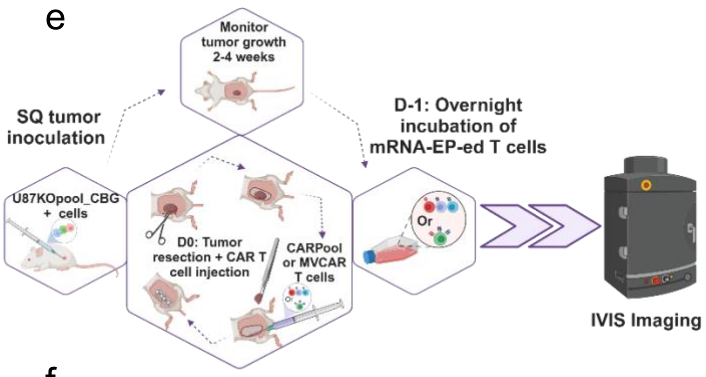


f

| Normal Donor | Sample | CAR stain (F(ab')$_2$_SA-APC) | | rhEphA2-HisTag-Pac. Blue | |
|---|---|---|---|---|---|
| | | % CAR-APC | MFI (CAR-APC) | % rhEphA2 | MFI (rhEphA2) |
| #1 | EphA2#1 CAR | 93.8 | 18484 | 78.1 | 14440 |
| #1 | EphA2#2 CAR | 81.9 | 4905 | 46.6 | 2462 |
| #1 | MVCAR#1 | 66 | 4214 | 24.2 | 2424 |
| #1 | MVCAR#2 | 75.3 | 5899 | 21.1 | 2058 |
| #2 | EphA2#1 CAR | 93.4 | 3515 | 73.5 | 2177 |
| #2 | EphA2#2 CAR | 88.7 | 2252 | 32.1 | 1471 |
| #2 | MVCAR#1 | 96 | 9693 | 78 | 2120 |
| #2 | MVCAR#2 | 94.9 | 6565 | 21 | 1300 |

**Fig. S5. Investigating the influence of conditioned media on the cytotoxicity of LNP-mediated mRNA-based MVCAR. (a-d):** MVCAR#2_LNP cells expanded in either ImmunoCult$^{TM}$-XF plus IL-2 or (IL-7+IL-15), were prepared by adding 1ug encapsulated mRNA per 1e6 cells or 3ug per 1e6 cells, respectively. 24 hrs later, MVCAR#2_LNP were cocultured with U87KOpool-CBG-GFP/NLS target cells at 10:1 effector-to-target ratios and monitored in real time by running the imaging module on the RTCA eSight machine. Normalized green fluorescent Intensities were then used to calculate % cytolysis at each time point. N= 3 biological replicates from one donor. (**b** and **d**): One-way ANOVA followed by Dunnett's multiple comparison analysis). (**e**): Workflow for running the resection NSG SQ GBM mouse model involving mRNA-based CAR T cell locoregional injections. (**f**): Table showing comparison between the binding capacity and MFI obtained by flow cytometry staining of EphA2#1 or EphA2#2 CAR T cells with rhEphA2-HisTag and anti-HisTag-Pacific Blue in HER2 CAR T cells or MVCAR (n= 2 separate donors). CAR staining was performed by staining with biotin-SP-conjugated affiniPure F(ab')$_2$ fragment goat anti-human IgG and streptavidin-APC (SA-APC).

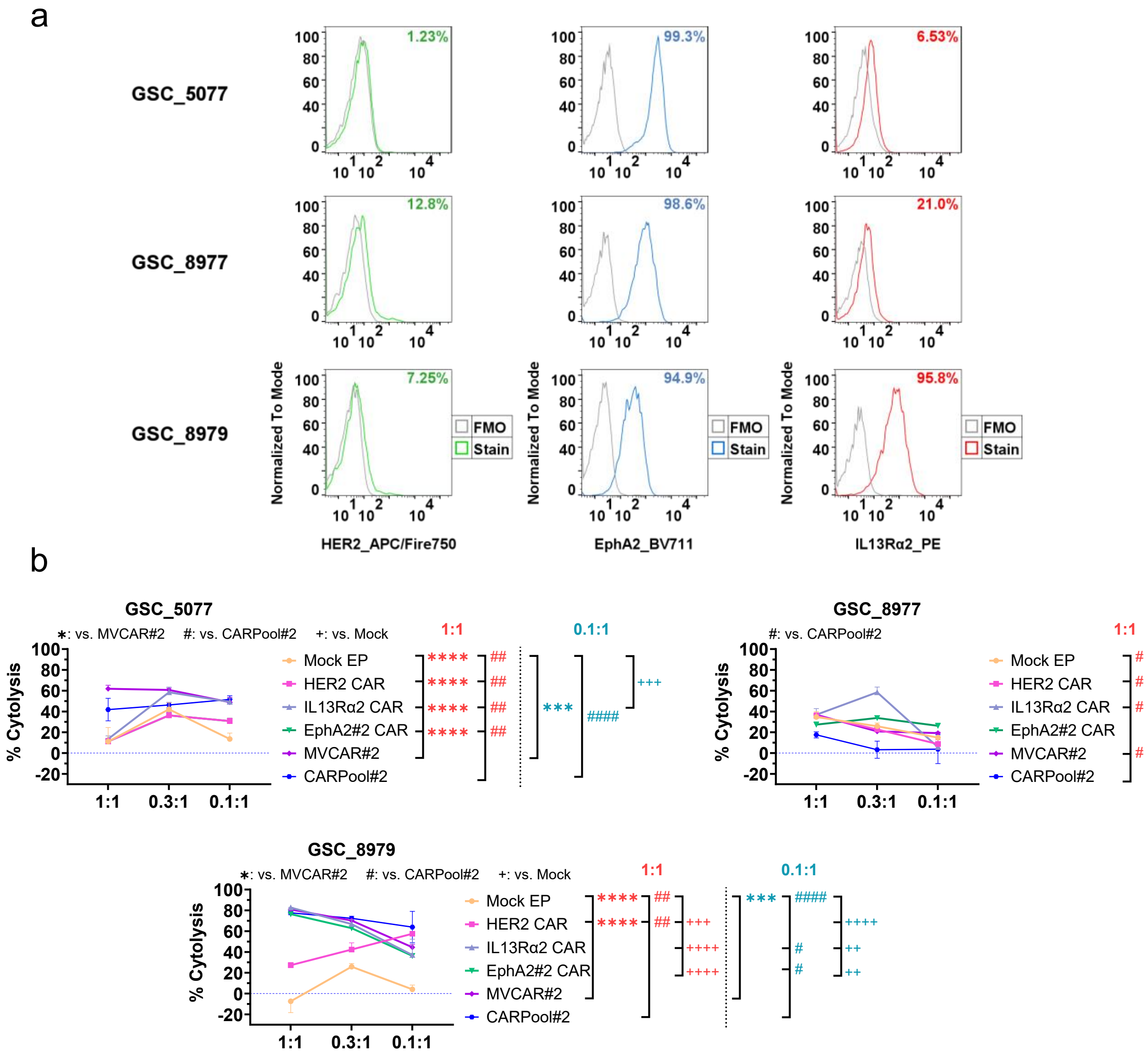


**Fig. S6. Cytotoxic efficacy of mRNA-based CAR T cells against patient-derived GSCs.** (**a**): Three patient-derived GSCs (#5077, #8977, and #8979) were phenotypically evaluated by flow cytometry for the cell surface target expression (of HER2, IL13Rα2, and EphA2) as compared to FMO. (**b**): Mean ± SD of % cytotoxicity of single-targeting mRNA-based CAR T cells, CARPool#2, or MVCAR#2 at multiple E:T ratios (n= 1 healthy donor, 3 biological replicates per sample; two-way ANOVA, post hoc Tukey).

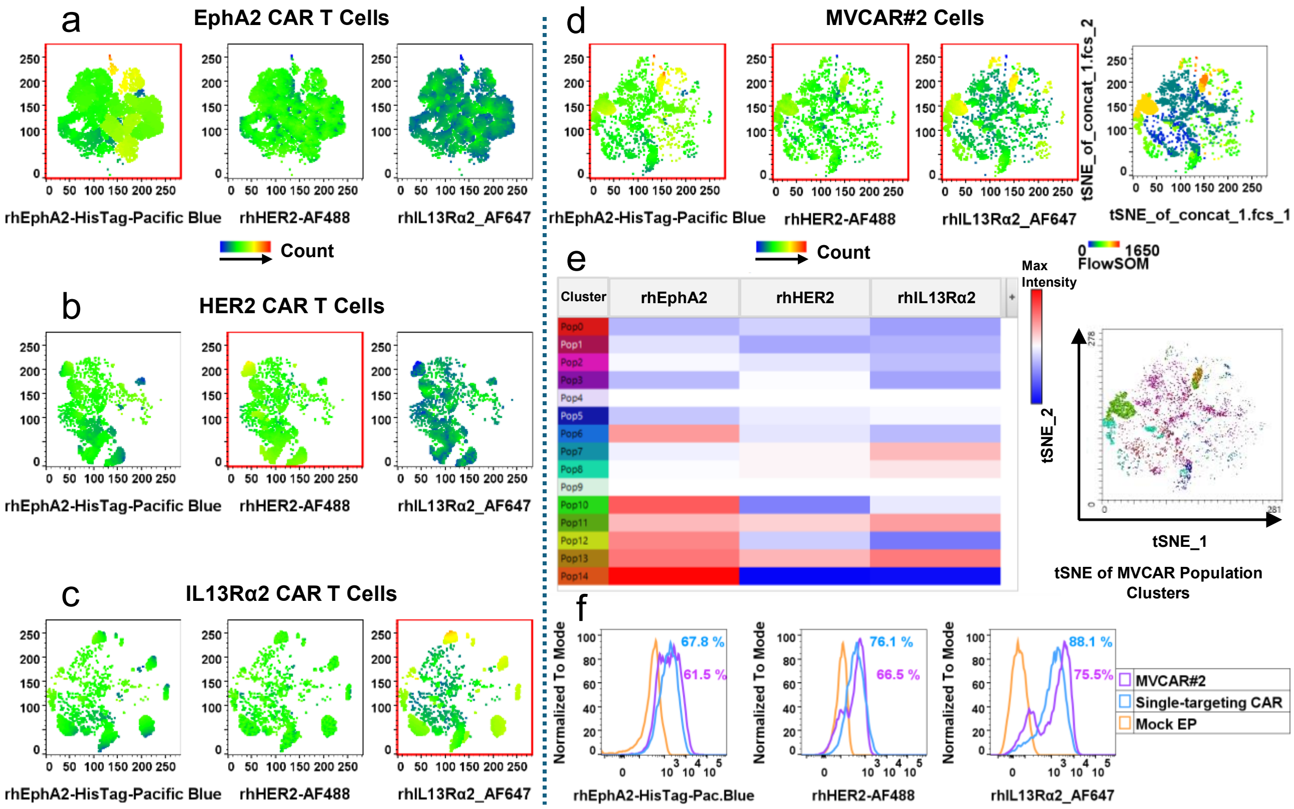


**Fig. S7. Population diversity in MVCAR T cells.** (**a-f**): Representative flow cytometry staining of CAR T cells individually EP-ed to generate single targeting CAR T cells (**a-c**), or EP-ed simultaneously with mRNA encoding for HER2 CAR, IL13Rα2 CAR, and EphA2#2 CAR (1:1:1.1 µg per 1e6, respectively) to generate MVCAR#2 (**d, e**). MVCAR#2 cells were co-stained with rhChimera of rhEphA2, rhHER2, and rhIL13Rα2, analyzed by FlowJo®, and plotted as tSNE plots. (**d, e**): Heatmap and tSNE plots generated by FlowSOM show at least 12 different populations of distinct CAR combinations expressed in MVCAR#2 cells. (**f**): Histograms demonstrating percent staining with rhChimera in single CAR T cells versus MVCAR#2.
